# CeO_2_ Supported Gold Nanocluster Catalysts for CO Oxidation: Surface Evolution Influenced by the Ligand Shell

**DOI:** 10.1002/cctc.202200322

**Published:** 2022-05-18

**Authors:** Vera Truttmann, Hedda Drexler, Michael Stöger‐Pollach, Tokuhisa Kawawaki, Yuichi Negishi, Noelia Barrabés, Günther Rupprechter

**Affiliations:** ^1^ Institute of Materials Chemistry TU Wien Getreidemarkt 9/165 1060 Vienna Austria; ^2^ University Service Center for Transmission Electron Microscopy (USTEM) TU Wien Wiedner Hauptstraße 8–10 1040 Vienna Austria; ^3^ Department of Applied Chemistry Faculty of Science Tokyo University of Science Kagurazaka, Shinjuku-ku Tokyo 162-8601 Japan

**Keywords:** CO oxidation, heterogeneous catalysis, *in situ*/*operando* infrared spectroscopy, ligand effect, metal nanoclusters

## Abstract

Monolayer protected Au nanocluster catalysts are known to undergo structural changes during catalytic reactions, including dissociation and migration of ligands onto the support, which strongly affects their activity and stability. To better understand how the nature of ligands influences the catalytic activity of such catalysts, three types of ceria supported Au nanoclusters with different kinds of ligands (thiolates, phosphines and a mixture thereof) have been studied, employing CO oxidation as model reaction. The thiolate‐protected Au_25_/CeO_2_ showed significantly higher CO conversion after activation at 250 °C than the cluster catalysts possessing phosphine ligands. Temperature programmed oxidation and *in situ* infrared spectroscopy revealed that while the phosphine ligands seemed to decompose and free Au surface was exposed, temperatures higher than 250 °C are required to efficiently remove them from the whole catalyst system. Moreover, the presence of residues on the support seemed to have much greater influence on the reactivity than the gold particle size.

## Graphical abstract

**Heterogeneous CO oxidation**: A ligand effect was observed in heterogeneous CO oxidation by Au nanoclusters after oxidative pretreatment at 250 °C, with thiolate‐protected clusters showing significantly higher conversion than phosphine‐protected clusters, for which ligand residues blocked the active interfacial sites.

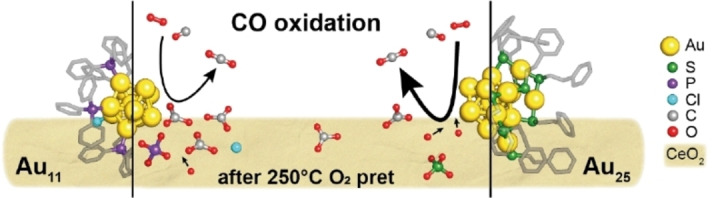

## Introduction

Heterogeneous catalysis employing nanomaterials is a well‐established field, often featuring metal nanoparticles supported on oxides.^[1]^ Among them, Au nanoparticles have been frequently used,[^1^, ^2^] especially since Haruta and coworkers reported their high activity in low‐temperature CO oxidation.^[3]^ As bulk gold is unreactive, the difference in activity was attributed to the small size and electronic structure of these nanoparticles.[^2a^, ^4^]

More recently, ligand protected Au nanoclusters immobilized on various supports have also been applied in heterogeneous catalysis.^[5]^ Unlike their nanoparticle counterparts, Au nanoclusters can be readily prepared monodisperse, i. e. possessing a uniform size and structure.[^5b^, ^5d^, ^5e^, ^5f^, ^6^] Their molecule‐like properties are influenced by a variety of factors, for example the number of metal atoms and their arrangement,^[6]^ the presence of dopant atoms[^6a^, ^7^] or their protecting ligands.^[8]^ For the latter, different classes can be employed,[^5a^, ^6b^] including for example thiolates[^5a^, ^6b^, ^8a^] or phosphines.[^5a^, ^9^] The ligands directly influence parameters such as cluster stability or polarity[^5a^, ^6b^, ^8a^, ^10^] and have therefore profound effect on the overall structural properties or catalytic activity.[^5a^, ^5c^, ^5d^, ^8a^, ^10^, ^11^] Modifying or replacing them by ligand exchange has become a useful tool for optimizing cluster properties.[^6a^, ^12^]

Combining versatility and high activity, Au nanoclusters can be used to catalyze different kinds of heterogeneous reactions,[^5a^, ^5b^, ^5d^, ^5f^] among which oxidations are the most studied.^[13]^ Due to their monodisperse nature and defined structure, they can be used as model systems, obtaining molecular level insight in the catalytic reaction.[^5a^, ^5b^, ^5d^, ^5e^, ^5f^, ^13^]

A variety of factors influence the performance of Au nanocluster catalysts in heterogeneous reactions: Probably the most evident is the number of metal atoms in the cluster, determining the structure. A size dependence of the catalytic activity – sometimes even of only a few atoms – has been reported for different types of reactions, for example, for CO,[^11a^, ^14^] cyclohexane^[15]^ or styrene oxidation.^[16]^ Furthermore, the catalytic behavior is influenced by the geometry[^5c^, ^11a^, ^17^] and heteroatom doping creating bimetallic nanoclusters.[^5f^, ^18^]

Previous work showed a strong effect of the support on the reactivity and stability of the cluster catalysts for various pretreatment conditions. For example, SiO_2_ supported Au nanoclusters showed higher activity in cyclohexane oxidation, whereas better selectivity was obtained using TiO_2_ as support material.^[15a]^ For CO oxidation, CeO_2_ supported Au nanoclusters were found to be significantly more active than those supported on Fe_2_O_3_,^[19]^ TiO_2_[^18b^, ^19^, ^20^] or Al_2_O_3_,^[20]^ related to ceria aiding the transfer of oxygen to CO adsorbed on Au sites.^[21]^ The shape of the CeO_2_ support particles is known to influence the CO oxidation activity of Au nanoclusters as well.^[22]^ Furthermore, the support material is also known influence the stability of nanocluster catalysts.[^14^, ^15^, ^22b^, ^23^]

To obtain optimal catalytic activity with cluster catalysts, the removal of ligands is essential to create accessible Au sites on the cluster surface.^[24]^ Oxidative pretreatment was found to significantly enhance the activity of a Au_25_/CeO_2_ in CO oxidation by Jin and coworkers.[^19^, ^24a^] Thereby, highest conversion was found for the sample pretreated at 250 °C for 1 h.^[24a]^ Similarly, Au_38_/CeO_2_ could be activated by oxidative thermal treatment at 175 °C for 2 h, while further increase of the pretreatment temperature resulted in reduced CO oxidation activity.^[25]^ However, combined oxidative and reductive treatment of Au_144_/CeO_2_ enhanced the catalytic performance, ascribed to the production of active oxygen species on the ceria support.^[26]^ Theoretical investigations of a Au_20_(SCH_3_)_16_ cluster on CeO_2_ showed that optimal cluster‐support interaction and O_2_ adsorption are achieved by partial ligand removal.^[27]^ Furthermore, the thiolate ligand desorption of a Au_25_(SC_12_H_25_)_18_/CeO_2_ catalyst for CO oxidation was also facilitated by adding water vapor to the pretreatment gas.^[28]^ All this clearly indicates the importance of catalyst activation by ligand removal from Au nanocluster catalysts.

Our recent studies of Au_38_/CeO_2_ catalysts by X‐ray absorption spectroscopy (XAS) revealed that the temperature induced rearrangements of the cluster structure during activation cannot be solely explained by detachment of thiolate ligands.^[29]^ While the ligands start to disintegrate already at 150 °C, the cluster surface is still covered by Au^+^−S units which can only be removed at higher temperature.^[29a]^ However, sulfur moieties remain in the system even after oxidative treatment at 250 °C.[^29^, ^30^]

Comparing the influence of different thiolate ligands on the CO oxidation activity of Au_*n*_/CeO_2_ catalysts (*n*=25, 36, 38), it was found that they determine the steric hindrance on the perimeter sites crucial for CO adsorption.^[11a]^ A distinct influence of the type of protecting thiolate ligand on the reactivity was also found for Au_28_(SR)_20_/CeO_2_ catalysts (R=cyclohexyl and 4‐*tert*‐butylphenyl).^[11c]^ The CO oxidation activity is therefore apparently influenced by the nature of the protecting ligands of a cluster catalyst as well.[^11a^, ^29^]

Nevertheless, the investigation of the “ligand effect” in Au nanocluster CO oxidation catalysis has so far mainly been focused on *thiolates* as protecting ligands. Phosphine‐protected Au nanoclusters have only rarely been employed as catalysts for this reaction. Wu *et al*. reported a highly active Au_22_(1,8‐bis(diphenylphosphino)octane)_6_ catalyst, where uncoordinated gold atoms were identified as the active sites.^[20]^ For a series of PPh_3_‐protected Au_*n*_ species (*n*=1, 8, 9, 101), mild thermal treatment (up to 120 °C) was found to alter the structure depending on the nature of the support material: Whereas fragmentation into small (Au−PPh_3_)^+^ units was observed on supports with mainly Brønsted acid sites such as SiO_2_, immobilizing Au clusters on Lewis acidic supports such as CeO_2_ resulted in exposure of the bare Au_*n*_ cores due to migration of the phosphine ligands. When testing the pretreated CeO_2_ supported Au_*n*_ catalysts in CO oxidation, activity was found to be strongly size‐dependent, with the larger clusters being more active.^[14]^ All these studies support the hypothesis that the ligands of the Au nanoclusters play a significant role for catalysis.

**Figure 1:**
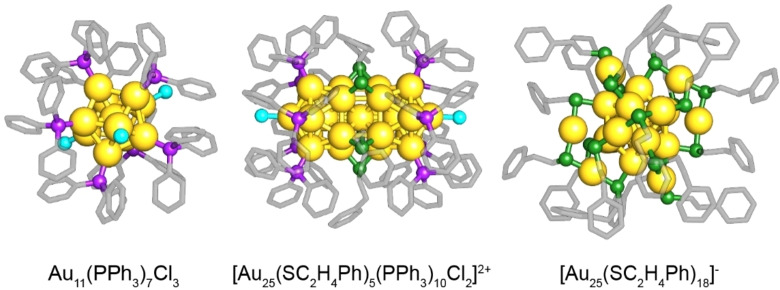
Structures of the three Au nanoclusters employed in this study; from left to right: Au_11_(PPh_3_)_7_Cl_3_, [Au_25_(SC_2_H_4_Ph)_5_(PPh_3_)_10_Cl_2_]^2+^ and [Au_25_(SC_2_H_4_Ph)_18_]^−^. Color code: Au=yellow, P=purple, Cl=cyan, S=green, C=grey. The images are based on structures determined by X‐ray crystallography.[^32^, ^33b^, ^34^]

Thus, herein the influence of the clusters’ initial ligand shell on the performance of Au nanoclusters in CO oxidation was studied. Three differently sized gold nanoclusters protected by different kinds of ligands were chosen as heterogeneous catalysts: phosphine‐protected Au_11_, thiolate‐protected Au_25_, and biicosahedral Au_25_ with a mixed phosphine/thiolate ligand shell. The cluster structures are illustrated in Figure 1. All three clusters, Au_11_(PPh_3_)_7_Cl_3_, [Au_25_(SC_2_H_4_Ph)_5_(PPh_3_)_10_Cl_2_]^2+^ and the anionic [Au_25_(SC_2_H_4_Ph)_18_]^−^, belong to the so‐called “magic number series”, indicating that they are very stable because of having closed electron shells.^[31]^ Furthermore, they can be considered “standard clusters” in their respective class.[^9^, ^31b^, ^32^] The biicosahedral cluster [Au_25_(SC_2_H_4_Ph)_5_(PPh_3_)_10_Cl_2_]^2+^ is straightforwardly prepared by treating Au_11_(PPh_3_)_7_Cl_3_ with an excess of 2‐phenylethanethiol (2‐PET) in solution and represents an intermediate between fully phosphine or thiolate protected clusters.^[33]^ Motivated by previous studies, CeO_2_ was used as support material since it leads to high CO oxidation activity while stabilizing the cluster structure at elevated temperatures.[^14^, ^19^]

Pretreatment studies showed that heating to 250 °C under oxidative atmosphere is sufficient for activation of Au_25_/CeO_2_, while 300 °C are required for Au_11_/CeO_2_ and Biico Au_25_/CeO_2_. This appears to be related to the process of ligand removal, necessary to produce accessible Au surface for heterogeneous catalysis. Furthermore*, in situ* infrared measurements of the Au_*n*_/CeO_2_ catalysts were performed, allowing to obtain an understanding of ligand behavior upon pretreatment and reaction. IR bands of the thiolate and/or phosphine ligands clearly decreased during oxidative pretreatment, whereas several bands related to adsorbed species were formed. CO adsorption experiments after pretreatment showed that the activation process yielded exposed Au surfaces for all three cluster catalysts. Reduction of Au^+^ species was observed during reaction, which was more efficient for Au_25_/CeO_2_ compared to the other catalysts. Thus, the significant differences in catalytic activity might be related to ligand residues located on the support rather than on the Au particles, potentially blocking crucial interfacial sites.

## Results and Discussion

The three types of gold nanoclusters were prepared and purified as described in the Supplementary Material. Characterization of the unsupported clusters was performed by Ultraviolet‐visible spectroscopy (UV‐Vis), attenuated total reflection infrared spectroscopy (ATR‐IR), and matrix‐assisted laser desorption/ionization (MALDI) or electrospray ionization mass spectrometry (ESI‐MS), confirming the purity of the samples. The supported catalysts were prepared by wet impregnation of ceria, yielding a Au loading of 1.2 wt%. Refer to the Supplementary Material for further details.

### Effect of the Pretreatment Temperature

Catalyst activation, prior to CO oxidation, is closely linked to (partial) removal of the respective ligand monolayer from the nanoclusters.[^5a^, ^24a^, ^24b^, ^29a^] The optimal conditions were thus determined for each cluster‐ligand configuration. Based on previous work,[^15a^, ^29a^] thermal oxidative pretreatment (5 % O_2_ in Ar) was chosen for this step, with the maximum temperature varied from 150 °C to 300 °C and held for 30 minutes.

**Figure 2:**
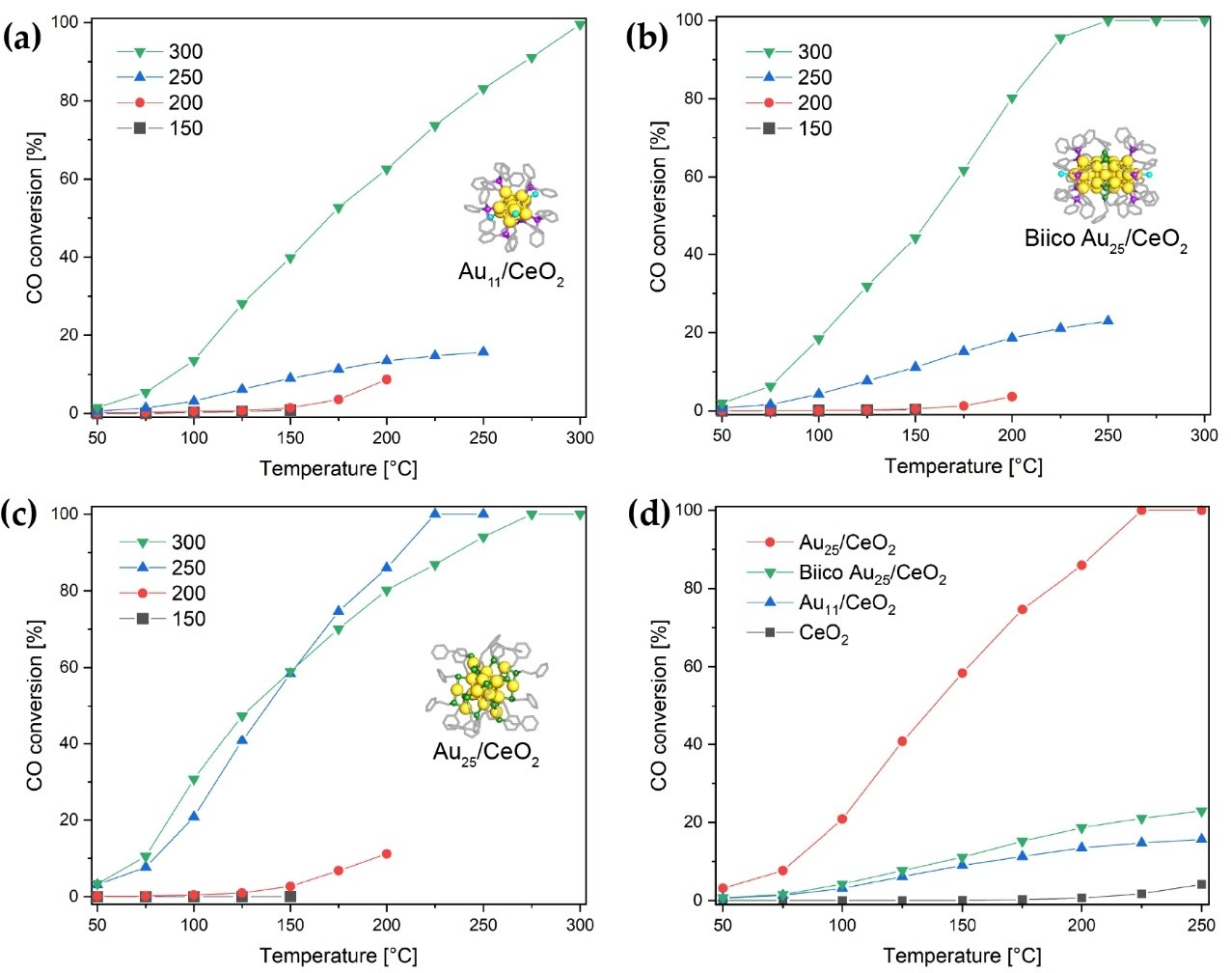
Catalytic activity of Au nanoclusters on CeO_2_ (0.3 wt% Au loading, 15 mg catalyst) in CO oxidation depending on the temperature of oxidative pretreatment: (a) Au_11_/CeO_2_; (b) Biico Au_25_/CeO_2_; (c) Au_25_/CeO_2_. Comparison of the catalytic activity of the different nanocluster catalysts pretreated at 250 °C (d).

As seen in Figure 2a–c, none of the samples showed significant activity after pretreatment at 150 °C. Similarly, only minor CO conversion above 150 °C was achieved with a 200 °C pretreatment. After pretreatment at 250 °C, Au_25_/CeO_2_ showed a sudden onset in activity, forming CO_2_ already at room temperature and reaching 100 % conversion above 200 °C (Figure 2c). Because Au_25_/CeO_2_ showed such a high activity, the Au loading was reduced to 0.3 wt% for all catalysts by further dilution with ceria. This enabled more meaningful measurements of the temperature‐dependent activity.

A similar pretreatment effect was observed for Au_38_/CeO_2_ previously, which showed significantly higher activity when activated at 250 °C than at 150 °C.^[29a]^ In contrast, Nie *et al*.^[19]^ observed that oxidative pretreatment at 150 °C for 1.5 h seemed to be optimal, considering that no further increase in CO conversion could be achieved at a pretreatment temperature of 250 °C. The same study reported that the duration of the pretreatment plays a significant role: 30 minutes pretreatment at 150 °C was considerably less effective for catalyst activation than 1.5 h. This might explain this difference.

An increase in activity after 250 °C pretreatment was also noted for Au_11_/CeO_2_ (Figure 2a) and Biico Au_25_/CeO_2_ (Figure 2b). However, as Figure 2d shows, these two cluster catalysts showed significantly lower CO conversion than the Au_25_/CeO_2_ sample pretreated at the same temperature. When the maximum temperature of the oxidative pretreatment was raised to 300 °C, all three catalysts had high activity in CO oxidation. Moreover, even though there were differences in conversion levels of the three catalysts, these were less pronounced as after pretreatment at 250 °C (see Figure S7c), indicating that the activation of the Au nanoclusters strongly depends on their specific ligand. It seems that phosphine ligands hinder catalyst activation at and below 250 °C. Since the greatest difference in catalytic activity of the cluster catalysts was observed at 250 °C, it was chosen as pretreatment and reaction temperature for all further studies.

Potential catalyst deactivation after pretreatment at 250 °C was studied by performing three consecutives runs with each sample. A sample was cooled to room temperature in inert gas atmosphere after reaching 250 °C reaction temperature and then the reaction was carried out two more times (without further pretreatment). As Figure S9 shows, each catalyst only showed minor signs of deactivation or activation.

### Modifications by Oxidative Pretreatment

Changes of the cluster catalysts imposed by oxidative pretreatment were further studied by temperature programmed oxidation (TPO), thermogravimetric analysis (TGA)/differential scanning calorimetry (DSC) and *in situ* transmission infrared measurements. Based on their similar conversion levels at these loadings after pretreatment at 250 °C, 1.2 wt% Au_11_/CeO_2_ and 1.2 wt% BiicoAu_25_/CeO_2_ were compared to 0.3 wt% Au_25_/CeO_2_.

**Figure 3:**
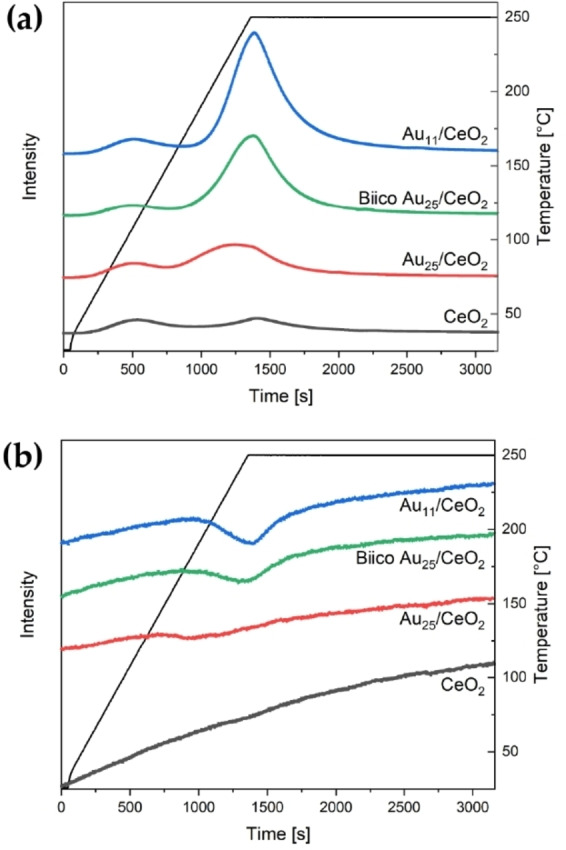
CO_2_ generation (a) and O_2_ consumption spectra (b) of the different catalysts during oxidative pretreatment until 250 °C. Au content in catalyst: Au_11_ and BiicoAu_25_/CeO_2_: 1.2 wt%, Au_25_/CeO_2_: 0.3 wt%. Spectra were normalized by the carrier gas signal to compensate for changes in pressure. Spectra are offset for better visibility.

In a first step, the approximate ligand decomposition/desorption temperature was estimated by studying the CO_2_ generation and O_2_ consumption mass spectra (Figure 3) during pretreatment (performed in the *in situ* infrared cell). The relatively low intensity of the Au_25_/CeO_2_ signals in Figure 3 is due to the lower Au content. This was done to ensure that the activity of all three cluster catalysts was in a similar range for the *operando* infrared experiments (see later).

As shown in Figure 3a, low‐temperature generation of CO_2_ was observed from ∼60 °C onwards for all catalysts including pure CeO_2_, which is due to desorption of CO_2_ adsorbed on ceria at room temperature.^[19]^ Interestingly, the on‐set of ligand decomposition/desorption from the catalyst (marked by O_2_ consumption and CO_2_ evolution) varies significantly for the three clusters: For Au_25_/CeO_2_, CO_2_ generation and O_2_ consumption started at 150–155 °C, with a maximum at approximately 235 °C, which is in good agreement with Nie *et al*.^[19]^ Biico Au_25_/CeO_2_ and Au_11_/CeO_2_ showed evolution of CO_2_ only above 185 °C. For the latter two, the maximum CO_2_ formation was observed during the holding period at 250 °C. An additional TPO experiment until 300 °C finally showed that for these two clusters, the maximum is observed between 250 °C and 300 °C (Figure S10). The pure ceria support showed a minor CO_2_ signal above ∼200 °C. For all three cluster catalysts, CO_2_ generation and O_2_ consumption were observed in the same temperature range, clearly indicating oxidative removal of the organic protecting ligands. Note that the O_2_ consumption signal of Au_25_/CeO_2_ appears to be very weak, which is in fact caused by the reduced Au loading compared to the other catalysts, as well as the broadness of the peak.

TGA and DSC of the unsupported nanocluster samples (Figures S4–S6) further showed that while all three clusters exhibited mass loss up to at least 300 °C, Au_25_ was the only cluster with DSC features just below 250 °C, likely indicating that potential structural changes are already completed at 250 °C. However, it is unclear if the same changes also happen for supported clusters or if they would adapt different geometries upon supporting.

This may also explain the different activity of the nanocluster catalysts at 250 °C: In case of Au_25_/CeO_2_, due to an earlier on‐set of the ligand removal, the Au/oxide interfacial sites are better accessible, leading to significantly higher activity than that of the phosphine‐protected cluster catalysts. Rising the pretreatment temperature to 300 °C is sufficient to remove also (most of) the ligands of the latter, diminishing the differences in activity (see Figure S7c).

**Figure 4:**
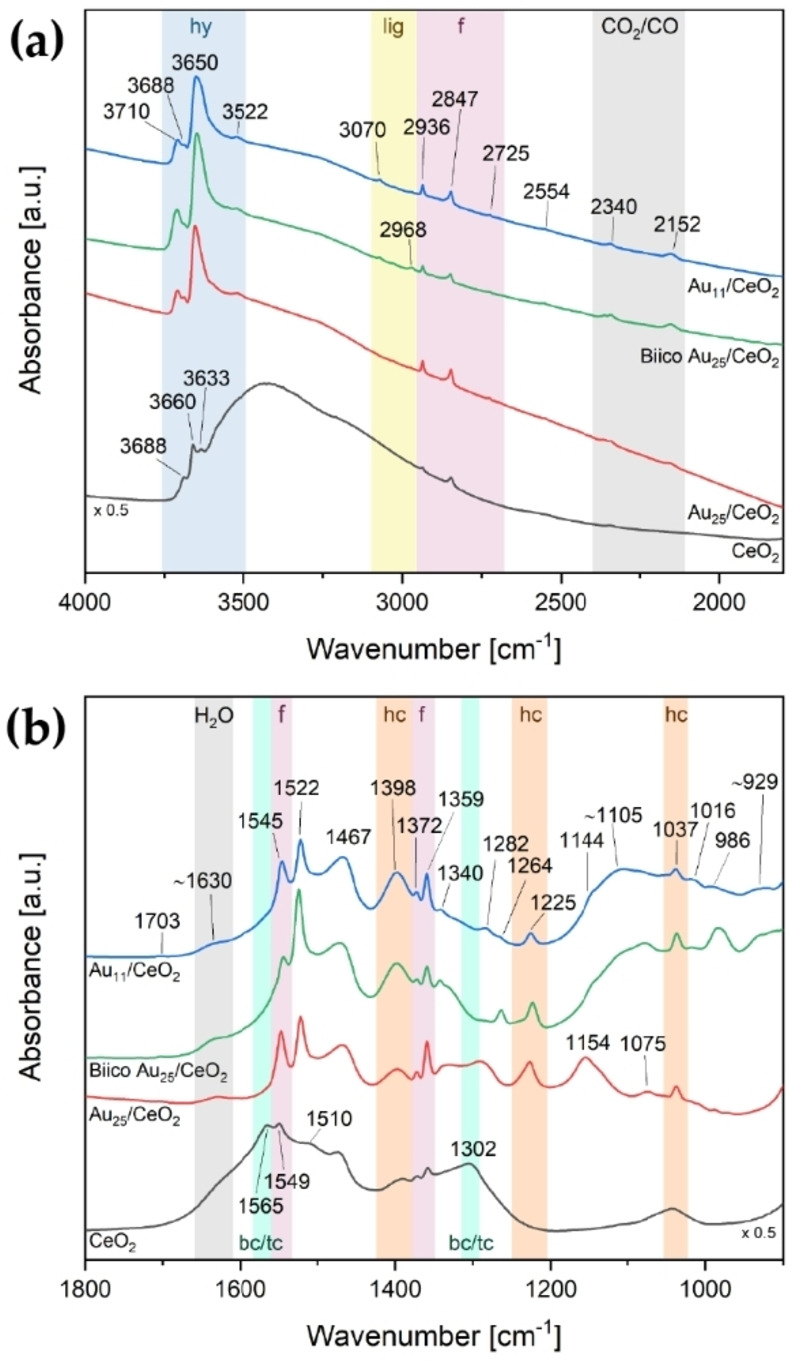
Infrared spectra of the catalysts after oxidative pretreatment at 250 °C: 4000–1800 cm^−1^ (a) and 1800–900 cm^−1^ (b). Bands associated with certain species are highlighted: hy=hydroxy species (blue), lig=ligand/other organic residues (yellow), f=formates (red), CO_2_/CO/H_2_O (grey), bc/tc=bidentate/tridentate carbonates (turquoise), hc=hydrogen carbonates (orange). Spectra are offset for better visibility and the spectrum of CeO_2_ was multiplied with 0.5 to allow for comparison with the cluster catalysts. Au content in catalyst: Au_11_ and BiicoAu_25_/CeO_2_: 1.2 wt%, Au_25_/CeO_2_: 0.3 wt%.

**Figure 5:**
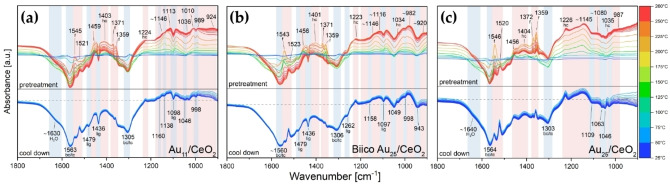
Difference spectra of the cluster catalysts during oxidative pretreatment: (a) Au_11_/CeO_2_, (b) Biico Au_25_/CeO_2_ and (c) Au_25_/CeO_2_. Bands decreasing during the pretreatment are indicated by a light blue background color and marked at the bottom, increasing ones by a light red one and marked on top. Assigned species are indicated by abbreviations: f=formates, hc=hydrogen carbonates, bc/tc=bidentate/tridentate carbonates, lig=ligands. For all samples, the spectra of the as‐prepared catalysts in He at RT were used as background. Au content in catalyst: Au_11_ and BiicoAu_25_/CeO_2_: 1.2 wt%, Au_25_/CeO_2_: 0.3 wt%. Difference spectra featuring the frequency region from 3800–2500 cm^−1^ and from 2500–2000 cm^−1^ can be found in the Supplementary Information (Figures S12–S14).

The 250 °C oxidative pretreatment of all catalysts was also followed by *in situ* transmission infrared spectroscopy. Figure 4 shows a comparison of the room temperature spectra after pretreatment and Figure 5 the difference spectra from 1800–900 cm^−1^ acquired during pretreatment. The following section will first discuss the final state obtained after pretreatment before dealing in detail with the significantly more complex *in situ* data of the pretreatment.

To get insights into the ligand‐support interaction and its evolution during pretreatment, one should focus on bands related to hydroxyl, formate and (hydrogen) carbonate species on CeO_2_ (e. g.^[35]^). A list of bands associated with these compounds is presented in the Supplementary Information in Table S1. However, typically several of these species coexist on the support and overlapping features make an accurate assignment difficult. Therefore, a thorough analysis of the pure supports is required for reference.

Pretreated catalysts (Figure 4) displayed formate related bands at 2936, 2847, 2725, 1565 (low intensity; only visible in the spectrum of pure CeO_2_), 1545, 1372 and 1359 cm^−1^.^[35]^ Hydrogen carbonate species were observed at 1398, 1225 and 1037 cm^−1^.[^35a^, ^36^]

For all samples including the plain support without clusters, intense negative bands were observed in the pretreatment difference spectra (Figure 5a–c and Figure S15c; as‐prepared catalysts in He at RT used as background) at 1565–1560 and 1306–1303 cm^−1^. These are related to the dissociation of either bidentate[^35b^, ^36^, ^37^] or tridentate[^35a^, ^37^] carbonate species upon temperature increase. The negative shoulder at ≈1630 cm^−1^ corresponds to the H_2_O bending vibration.^[35b]^ Simultaneously, bands were starting to appear 1459–1456 and 1404–1400 cm^−1^, followed by the formate bands (1546–1545, 1372–1371 and 1359 cm^−1^) and a sharp feature at 1523–1520 cm^−1^ (Figure 5a–c and Figure S15c). These bands at 1459–1456 and 1404–1400 cm^−1^ may indicate formation of polydentate carbonates,[^35^, ^37b^] presumably related to decomposition of the organic ligand framework. The band around 1400 cm^−1^ might also be associated with the formation of hydrogen carbonates.[^35a^, ^36^]

There were also changes in the higher wavenumber region (Figures S12a‐S15a): Removal of H_2_O is evidenced by the reduction of the broad band associated with O−H stretching vibrations (approximately 3300 cm^−1^).^[38]^ However, as Figure 4a shows, bands at 3710, 3688, 3650 and 3522 cm^−1^ associated with hydroxylated species^[35]^ were still present in the catalyst samples after pretreatment. Besides the aforementioned bands associated with formate species (2936, 2847, 2725 cm^−1^), the Au_11_/CeO_2_ and the Biico Au_25_/CeO_2_ samples also showed further absorption features in the C−H stretching region (at 3070 and 2968 cm^−1^; blue and green curve in Figure 4a), which indicates that not all hydrocarbon species (adsorbates/ligand residues) were removed by the pretreatment.

Differences among the samples were especially pronounced in the region below 1300 cm^−1^. The purely thiolate ligand protected cluster Au_25_/CeO_2_ showed formation of bands in the difference spectrum (Figure 5c) at 1226, ≈1145, ≈1080, 1035 and 987 cm^−1^, as well as negative bands at 1109, 1063 and 1046 cm^−1^. Several of those were also identified in the difference spectra of CeO_2_ without clusters (1223, 1109, 1085, 1063, 1046, 1037 and 984 cm^−1^, see Figure S15c), indicating that these might be related to adsorbed (hydrogen) carbonate species on the support. The broad band formed at around 1145 cm^−1^, which is not present in the support spectra, could be related to the formation of sulfate species due to the oxidative decomposition of the thiolate protecting ligands.^[39]^ Previous studies have already shown that residues of thiolate ligands can still be present on the oxide support even after pretreatment at 250 °C, forming sulfate^[29]^ or sulfide species.^[40]^ However, it cannot be excluded that carbonate species are responsible for the IR bands in this area.^[35a]^

The removal of the hydrocarbon framework of the ligands of Au_25_/CeO_2_ can be confirmed by IR spectroscopy: The difference spectrum in Figure S14a shows a negative band at 2926 cm^−1^, which can be attributed to the most intense C−H stretching vibration of the 2‐PET ligands (see Figure S3c for reference). Moreover, while the absorption spectrum of the as‐prepared catalyst still shows weak bands at 3060, 3027 and 2925 cm^−1^ related to the ligands, these are not present anymore for the pretreated catalyst (Figure S22a).

The difference spectra acquired during pretreatment of the phosphine‐protected clusters, Au_11_/CeO_2_ and Biico Au_25_/CeO_2_, depicted in Figure 5a–b and Figures S12‐S13, clearly show the partial removal of intact ligands (negative bands at 3055, 1479, 1436 and 1098 cm^−1^ for Au_11_/CeO_2_ and at 3060, 2926, 1479, 1436, 1262 and 1097 cm^−1^ for Biico Au_25_/CeO_2_). For both of them, the range from 1300–900 cm^−1^ is very complex due to appearing broad absorption features. Dai *et al*.^[41]^ showed that PO_*x*_ on CeO_2_ gives rise to IR bands at approximately 1158, 1000 and 950 cm^−1^. However, the bands appeared very broad and undefined, especially for lower PO_*x*_ contents. Thus, formation of phosphate species on the CeO_2_ support could be a potential explanation of this considerable increase in absorbance in this region, especially considering that this was only observed for the P‐containing cluster catalysts. Once again, however, it should be noted that also IR bands of carbonate species can be found in this wavenumber range,^[35a]^ and might also cause the bands observed for these samples, pointing to a combination of both phosphates and carbonates. Nevertheless, compared to Au_25_/CeO_2_ and CeO_2_, these two catalysts show a significant increase in IR absorbance below 1200 cm^−1^. Independent of its exact origin, this strongly indicates the presence of adsorbed species, which may influence the catalyst activity.

**Figure 6:**
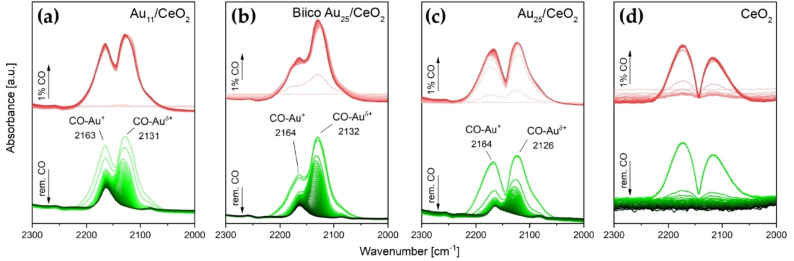
Transmission infrared spectra of room temperature CO adsorption on catalysts after oxidative pretreatment at 250 °C: (a) Au_11_/CeO_2_ (b) Biico Au_25_/CeO_2_, (c) Au_25_/CeO_2_ and (d) CeO_2_. The red spectra were obtained during exposure of the sample to an atmosphere of 1 % CO in He (50 ml/min total gas flow), the green spectra upon removal of gas phase CO by flowing 50 ml/min He. Au content in catalyst: Au_11_ and BiicoAu_25_/CeO_2_: 1.2 wt%, Au_25_/CeO_2_: 0.3 wt%.

The *in situ* IR measurements also showed that the hydrocarbon framework of the ligands for all three cluster catalysts is at least partially removed from the overall catalytic system, evidenced by significant formation of gas phase CO_2_ during pretreatment (see difference spectra of the catalysts in Figures S12b‐S14b). In addition, a band related to adsorbed CO was formed at ≈2150 cm^−1^, for all three. To probe the CO adsorption capability of the pretreated catalysts, 1 % CO in He was flown through the cell at room temperature until no further change was observed in the spectra. Afterwards, 100 % He was used to remove the CO atmosphere. As shown in Figure 6a–c, all three catalysts displayed a band of CO adsorbed on Au^δ+^ at roughly 2130 cm^−1^, which agrees with previous studies of Au nanoclusters.[^18b^, ^22^, ^29a^] For all three, flowing enough He through the cell after CO exposure results in a fully vanishing band. Nevertheless, since these species are believed to be the main active sites for CO oxidation on Au nanoclusters, their presence is highly important.^[24a]^

Furthermore, an additional band evolved with a maximum around 2164 cm^−1^, which remained after removal of the CO atmosphere. This band was attributed to CO on oxidized Au sites[^22b^, ^24a^, ^42^] and may be explained by some of the Au atoms in ligand‐protected nanoclusters bearing a positive charge.[^31a^, ^43^] Moreover, density functional theory (DFT) calculations on gas phase Au nanoclusters suggested a charge transfer from Au to Ce, leading to partially oxidized Au species.^[44]^ It is possible that a similar phenomenon occurs during ligand detachment increasing interaction between the Au core and the ceria support.

Consequently, it seems that oxidative pretreatment at 250 °C causes the desorption and (partial) decomposition (forming adsorbed species such as carbonates) of the ligand sphere, with parts of it then being completely removed from the system as CO_2_. It is thus effective to provide free Au sites for CO adsorption/reaction for all three catalysts; only small differences in the frequency and relative intensity of CO adsorbed on the different Au cluster catalysts were noted. The main difference among them seems to be the presence of further adsorbed species on the support. This is presumably related to the incomplete removal of the ligand sphere, especially concerning the (partially) phosphine‐protected clusters. Both Au_11_/CeO_2_ and Biico Au_25_/CeO_2_ showed residual weak bands located in the wavenumber region typical of C−H stretching vibrations and development of broad bands below 1200 cm^−1^.

These findings are in line with a previous study by Longo *et al*.,^[14]^ who found that the phosphine ligands of Au_*n*_ nanoclusters (*n*=1, 8, 9, 101) supported on CeO_2_ seemed to migrate to the support at 120 °C. This was explained by the favorable interactions between the phosphine ligands and the Lewis acidic centers of the CeO_2_ support. Migration to and oxidation of phosphine ligands on the support has also been described for Au_*n*_ (*n*=8, 9, 11, 101) nanoclusters on TiO_2_ subjected to different activation treatments (e. g. calcination in vacuum or O_2_ atmosphere).^[45]^ The same phenomenon has also been reported by Li and coworkers for biicosahedral Au_25_ encapsulated in SiO_2_.^[40]^ Thus, phosphine ligands likely detached from the Au core and migrated to the ceria surface, blocking the cluster‐support interface. This should have profound influence on the CO oxidation activity of the catalysts, owing to the importance of the interfacial sites,^[46]^ especially in a Mars‐van Krevelen like mechanism.[^14^, ^24a^]

To remove the phosphine ligands, higher temperatures are required than for thiolate ones, contrary to what is expected taking into account the difference in bond strength (Au−S>Au−P).[^10^, ^43a^] Thus, the ligand interaction with the support must be considered as well.

### *Operando* Infrared Studies of CO Oxidation

**Figure 7:**
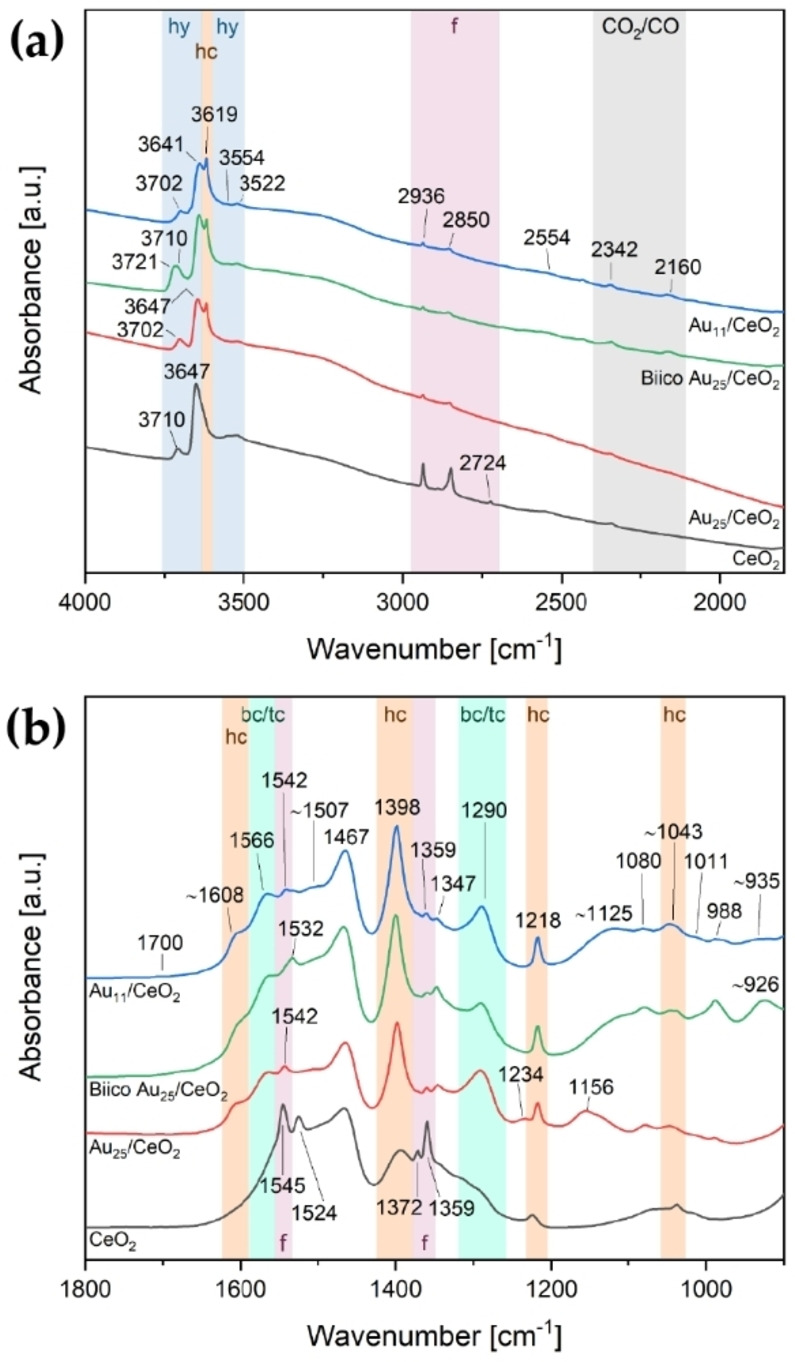
Infrared spectra of the catalysts after CO oxidation at 250 °C: 4000–1800 cm^−1^ (a) and 1800–900 cm^−1^ (b). Bands associated with certain species are highlighted: hy=hydroxy species (blue), f=formates (violet), CO_2_/CO (grey), bc/tc=bidentate/tridentate carbonates (turquoise), hc=hydrogen carbonates (orange). Spectra are offset for better visibility. Au content in catalyst: Au_11_ and BiicoAu_25_/CeO_2_: 1.2 wt%, Au_25_/CeO_2_: 0.3 wt%.

**Figure 8:**
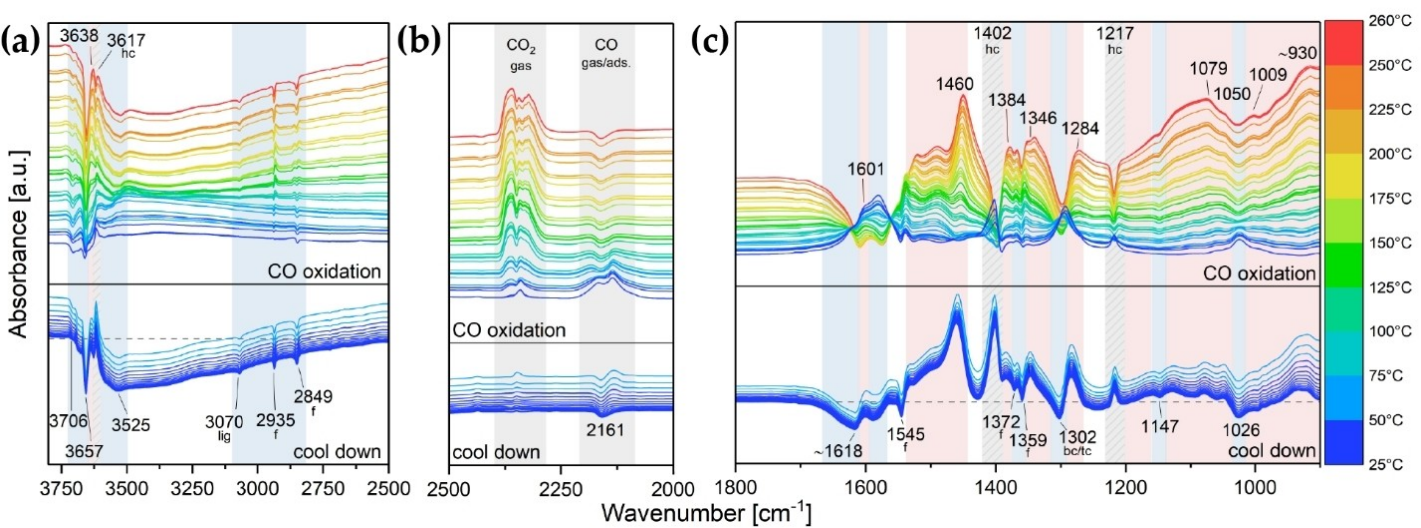
Difference spectra of Au_11_/CeO_2_ during CO oxidation: (a) 3800–2500 cm^−1^, (b) 2500–2000 cm^−1^ and (c) 1800–900 cm^−1^. Bands decreasing during the pretreatment are indicated by a light blue background color and marked at the bottom, increasing ones by a light red one and marked on top. Bands decreasing during reaction but reforming at cool down are indicated by a grey shaded background. The spectrum of the pretreated catalyst after the CO adsorption experiment in He at RT was used as background. Au content in catalyst: 1.2 wt%. Assigned species are indicated by abbreviations: f=formates, hc=hydrogen carbonates, bc/tc=bidentate/tridentate carbonates, lig=ligands. The difference spectra of Biico Au_25_/CeO_2_ and Au_25_/CeO_2_ during reaction can be found in Figure S16 and Figure S17, respectively.

To gain further insight into potential dynamics of the catalysts during CO oxidation at 250 °C, *operando* ^[47]^ infrared studies were performed. CO oxidation was carried out in a transmission IR cell with the catalyst pressed into a thin pellet and CO conversion was followed by gas chromatography (see Figure S11). Figure 7 first compares of the infrared spectra of the used catalysts and Figure 8 then displays difference spectra of Au_11_/CeO_2_ acquired during CO oxidation. The difference spectra of Au_25_/CeO_2_ and Biico Au_25_/CeO_2_, as well as of the support, are shown in the Supplementary Information (Figures S16–S18).

By following the bands of adsorbed species on the support, an understanding of the dynamics during the catalytic reaction can be obtained. Compared to the pretreated catalysts shown in Figure 4, the bands associated with formates (2936, 2850, 2724, 1545, 1372, 1359 cm^−1^) decreased for all three cluster catalysts, whereas an increase was noticed for the support. Moreover, the cluster catalysts also showed evolving bands at 1566 and 1290 cm^−1^, which have been previously assigned to bidentate[^35b^, ^36^, ^37^] or tridentate[^35a^, ^37^] carbonates. As evidenced in the difference spectra during catalytic CO oxidation (Figure 8 and Figures S16–S18), the hydrogen carbonate bands at 3619, 1398 and 1218 cm^−1^ seemed to disappear with increasing temperature during the reaction, but formed again during cool down. Compared to after pretreatment, slight shifts are noticed (1225→1218 cm^−1^ and 1037→1043 cm^−1^). Furthermore, as described by Vayssilov et al.,^[35a]^ an additional band at 3619 cm^−1^, as well as the shoulder at ≈1608 cm^−1^ could be identified after reaction. As shown in Figure 7a, hydroxy species were also still present after CO oxidation for all samples. These dynamics show that the clusters are capable of converting such adsorbed (intermediate) species to CO_2_, making them active CO oxidation catalysts.

During CO oxidation, a broad band centered at 1467 cm^−1^ further increased. When compared to spectra of theCeO_2_ support at different stages of the catalytic process (Figure S23), this particular band seemed to change in unison with another broad band at 1394 cm^−1^. Similar observations were also reported by other authors,^[37]^ who ascribed the bands to either mono‐ or polydentate carbonate species. In the spectra of the cluster catalysts, the lower energy band overlapped significantly with the intense hydrogen carbonate band at 1398 cm^−1^, limiting the assignment. However, formation of polydentate carbonates during CO oxidation seems reasonable.

After reaction, no bands related to ligands or their residues in the region of C−H stretching vibrations could be identified anymore for Au_11_/CeO_2_ and Biico Au_25_/CeO_2_ (blue and green curve in Figure 7a). Analogous to the pretreatment, the spectra of the used catalysts (Figure 7) still showed variations between the three cluster catalysts, whereas no significant differences could be observed in the difference spectra during CO oxidation (Figure 8 and Figures S16–S17). This implies that the changes between the clusters that occurred during the oxidative pretreatment were maintained during catalytic CO oxidation (i. e. the broad absorption features below 1200 cm^−1^ for Au_11_/CeO_2_ and Biico Au_25_/CeO_2_ and the band at ca. 1156 cm^−1^ for Au_25_/CeO_2_; see also Figure 4b).

The CO adsorption by the catalysts, however, was affected by the reaction. As Figure 8b shows, a negative band at 2161 cm^−1^ was observed at the end of the reaction (for all cluster catalysts; see also Figure S16b and Figure S17b), which indicates reduction of Au^+^ during the reaction. However, for Au_11_/CeO_2_ and Biico Au_25_/CeO_2_, a weak maximum could still be detected at 2160 cm^−1^ after reaction (Figure 7a) which suggests that some oxidized Au sites were still present after CO oxidation.

Upon repeating the room temperature CO adsorption experiment after reaction (same as after pretreatment), only CO adsorbed on partially oxidized Au^δ+^ was observed for all catalysts (Figure S19a–c). A comparison with the spectra of the pretreated catalysts (Figure 6a–c) shows that the maxima of the CO−Au band remained mostly unchanged. The CO−Au^+^ band of Au_11_/CeO_2_ and Biico Au_25_/CeO_2_ was unaffected by the adsorption and thus showed no signal in the CO adsorption difference spectra of the used catalysts.

Accordingly, the main changes in the infrared spectra during reaction can be attributed to adsorbed carbonate species on the support. These changes seem to occur for all cluster catalysts. The only significant difference among them is the evolvement of the Au sites: Only Au^δ+^ was detected for the used Au_25_/CeO_2_ catalyst, whereas the originally phosphine‐protected clusters Au_11_/CeO_2_ and Biico Au_25_/CeO_2_ still contained small amounts of oxidized Au^+^ species. At this point, it is an open question whether this is related to ligand residues blocking sites near or on the Au particles or not. However, the oxidation state of Au can certainly be considered important for adsorbate binding^[42]^ and thus also catalytic oxidation of CO, with Au^δ+^ being the main active sites in heterogeneous CO oxidation with Au nanoclusters.^[24a]^

Moreover, the assumption that oxidized phosphine species on the CeO_2_ support block interfacial sites rather than CO adsorption on the Au surface is affirmed by the high intensity of CO adsorbed on Au for both Au_11_/CeO_2_ and Biico Au_25_/CeO_2_ (using the intensity of the gas phase CO band as reference; Figure S19a–b). This relative intensity difference is considerably less for Au_25_/CeO_2_ (Figure S19c) due to lower Au loading on the catalyst (0.3 wt% compared to 1.2 wt% for Au_11_/CeO_2_ and Biico Au_25_/CeO_2_) to ensure comparable activities (see Section 6.1 in the Supplementary Information). However, the difference in maximum CO conversion of Au_11_/CeO_2_ and Au_25_/CeO_2_ in the *operando* IR study was less than 10 % (Figure S11a), suggesting that the degree of exposed Au surface was not a critical factor.

### Electron Microscopy of Used Catalysts

**Figure 9:**
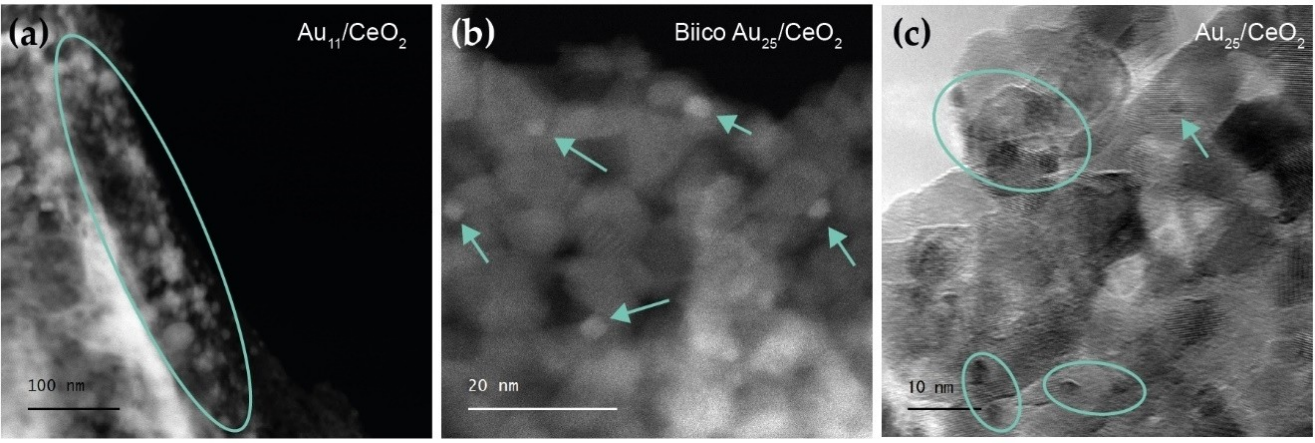
Electron microscopy images of the catalysts (1.2 wt% Au) after pretreatment and reaction at 250 °C: High‐angle annular dark‐field scanning transmission electron microscopy (HAADF‐STEM) of Au_11_/CeO_2_ (a) and Biico Au_25_/CeO_2_ (b); transmission electron microscopy (TEM) image of Au_25_/CeO_2_ (c). HAADF‐STEM images of Biico Au_25_/CeO_2_ and Au_25_/CeO_2_ showing a larger sample area can be found in Figure S24.

To determine the particle size of the Au clusters after reaction, (scanning) transmission electron microscopy ((S)TEM) images were taken. In line with previous studies, slight sintering of the Au clusters was observed after reaction. Figure 9 shows images of all three cluster catalysts after pretreatment and CO oxidation at 250 °C. Both Au_25_/CeO_2_ and Biico Au_25_/CeO_2_ mainly feature small particles with average sizes of ≈2.1 nm and ≈2.7 nm, respectively. Au_25_ clusters should be of ≈1.1 nm size.^[15a]^ Au_11_/CeO_2_, on the other hand, which should have a core diameter of 0.8 nm as a single cluster,^[48]^ appeared very polydisperse after reaction, but most particles still had sizes of roughly 4–7 nm. Migration of the phosphine ligands to the support during calcination and agglomeration of the bare Au particles was previously reported for Au_11_ on TiO_2_.^[45a]^

It is worth noting that the Au cluster size is not the predominant factor controlling catalyst activity in this study. Au_25_/CeO_2_ and Biico Au_25_/CeO_2_, both having particle sizes between 2–3 nm, show very different CO oxidation activity (see Figure 2d). In comparison, Biico Au_25_/CeO_2_ and Au_11_/CeO_2_ had similar CO conversion at a given temperature (Figure 2d), despite being significantly different in size and distribution on the surface. This confirms the suggestion that ligand residues and/or adsorbed species at the Au cluster‐support interfacial sites are causing the observed significant differences in catalytic activity.

## Conclusion

Three types of Au nanoclusters with different ligands and supported on CeO_2_ were examined. Depending on the cluster structure, type of ligand shell, and pretreatment, significant differences in the catalytic CO oxidation activity were observed. Whereas thiolate‐protected Au_25_/CeO_2_ reached 100 % conversion above 200 °C after a 250 °C pretreatment, the cluster catalysts containing phosphines in their ligand shell (Au_11_/CeO_2_ and Biico Au_25_/CeO_2_) exhibited only poor activity (below 30 % conversion at 250 °C). All samples showed stable conversion in three consecutives CO oxidation runs. After pretreatment at 300 °C, all catalysts exhibited comparable activity reaching 100 % above 250–300 °C. This seems related to differences in the catalyst activation process, as temperature programmed oxidation suggested that temperatures above 250 °C are required for oxidative removal of the ligands from Au_11_/CeO_2_ and Biico Au_25_/CeO_2_. Interestingly, this is contrary to the general concept of nanocluster stability, which classifies Au−S bonding stronger than Au−P. However, *in situ* infrared studies indicated that the ligands were mostly decomposed during pretreatment, as related bands decreased and CO adsorbed on Au could be observed after the activation process for all catalysts. In addition, a strong increase in absorbance below 1200 cm^−1^ was noticed for Au_11_/CeO_2_ and Biico Au_25_/CeO_2_, which may be related to formation of (oxidized) ligand residues on the support. Reduction of Au^+^ species was observed during CO oxidation and only IR bands related to CO adsorbed on Au^δ+^ were detected for the used Au_25_/CeO_2_ catalyst, whereas the others still additionally possessed a small amount of Au^+^ sites. It thus seems likely that fragments of the phosphine ligands and/or chlorines remained within the catalyst system of Au_11_/CeO_2_ and Biico Au_25_/CeO_2_, blocking active sites on the support‐Au interface and causing this striking difference in activity. No correlation was found between the size of the Au particles and the CO oxidation activity, owing to very different CO conversion of the similarly sized Au_25_‐based catalysts (2–3 nm after 250 °C reaction) and the negligible differences in activity between Au_11_/CeO_2_ and Biico Au_25_/CeO_2_ (4–30 nm and ≈2.7 nm, respectively). Consequently, the choice of a particular nanocluster and its ligands must be carefully considered in heterogeneous nanocluster catalysis, as each individual building block will affect the performance of the catalyst system.

## Experimental Section

**Catalyst Preparation**. The Au nanoclusters were synthesized and purified following published protocols.[^32^, ^33^, ^49^] The supported catalysts were prepared by wet impregnation of the clusters on CeO_2_. The gold loading of the catalysts was 1.2 wt%. Further details can be found in the Supplementary Material.

**Catalytic CO Oxidation Experiments**. Kinetic studies of the Au nanocluster catalysts in CO oxidation were pursued using a flow reactor coupled to a micro‐gas chromatograph (Micro‐GC, Fusion 3000 A, Inficon). ∼15 mg catalyst was placed between two glass wool plugs in a quartz glass tube, with a Ni/NiCr thermocouple submerged in the catalyst powder connected to a PID controller (EMSR EUROTHERM GmbH) of a cylindrical oven. All pretreatments were conducted in an oxidative atmosphere (5 % O_2_ in Ar, 50 ml/min total gas flow) with a temperature ramp of 10 °C/min. The maximum temperature (150 °C, 200 °C, 250 °C or 300 °C) was held for 30 minutes before cooling the sample to room temperature in Ar (50 ml/min). The gas flow composition was subsequently switched to reaction conditions (1 % CO and 2 % O_2_ in Ar; 50 ml/min total gas flow). The temperature was then increased to the respective maximum reaction temperature (the same as the maximum temperature of the pretreatment) with a ramp of 5 °C/min. From 50 °C onwards, every 25 °C, the temperature was kept constant for 10 min to allow for accurate conversion measurements by micro‐GC. After reaction, the catalyst was cooled to room temperature in argon (50 ml/min).

To investigate whether there was any deactivation or activation of the catalyst after pretreatment and reaction at 250 °C, three consecutive CO oxidation runs were conducted. Therefore, after pretreatment and the first run as described above, once the samples had cooled to room temperature, the gas composition was again changed to reaction conditions and the samples heated another time to 250 °C (same process as in the first run, no further pretreatment). This was then also repeated for a third time.

To compare the activity of the Au nanocluster catalysts, all data were normalized to 15 mg catalyst with a Au loading of 0.3 wt%. It should be noted that the CeO_2_ support shows only minor activity above 200 °C (see Figure S8).

***In situ*** **/*Operando* Transmission Infrared Studies**. *In situ/Operando* transmission Fourier‐transform infrared studies (transmission FTIR) were conducted using a Bruker Vertex 70 spectrometer. About 10 mg of catalyst were grinded thoroughly and pressed into a thin pellet using a hydraulic press. The pellet was then mounted in a flow cell with IR transmissible windows and a thermocouple connected to a PID controller. The product gas flow was analyzed by GC chromatography (HP‐PLOT Q column, FID detector) and mass spectrometry (Pfeiffer Vacuum, Thermostar).

The sample was pretreated as described in the previous section (10 °C/min to 250 °C, hold for 30 min, 5 % O_2_ in He, 50 ml/min total gas flow) while simultaneously recording IR spectra (MIR, resolution 4 cm^−1^).

After cooling to room temperature in helium, a CO adsorption experiment was performed. Therefore, the sample was exposed to 1 % CO in He (50 ml/min total flow) until the CO IR band did not change significantly anymore. Subsequently, 50 ml/min He was flown through the cell until no further significant changes were observed in the IR spectrum.

Following the CO adsorption experiment, the gases were switched to CO oxidation conditions (1 % CO, 2 % O_2_ in He, 50 ml/min total flow) and the reaction conducted as described in the previous section (heat up to 250 °C with 5 °C/min, hold every 25 °C for 10 min) while following by IR. The sample was then cooled to room temperature in He and the CO adsorption experiment repeated once more.

**Transmission Electron Microscopy (TEM) and High‐angle Annular Dark‐field Scanning Transmission Electron Microscopy (HAADF‐STEM)**. Electron microscopy was performed using a 200 kV FEI Tecnai F20 S‐TWIN analytical (scanning) transmission electron microscopy [(S)TEM] instrument equipped with a Gatan GIF Tridiem filter. The energy resolution was ≤1 eV, the semiconvergence angle ∼8 mrad, the semicollection angle ∼15 mrad, and the spatial resolution on the order of 0.5 nm. Supported clusters were directly deposited on carbon‐coated copper grids and plasma cleaning was applied to remove possible hydrocarbons and adsorbed water.

**Molecular Graphic Images**. Molecular graphics images were produced using the UCSF Chimera package^[50]^ from the Resource for Biocomputing, Visualization, and Informatics at the University of California, San Francisco (supported by NIH P41 RR001081).

## Author Contributions

Synthesis of the Au nanoclusters was performed by V.T. and T.K. IR, TPO, TGA/DSC and kinetic measurements and data evaluation were done by V.T. (S)TEM was measured by M.S.P. and ESI‐TOFMS by H.D. Final interpretation and manuscript preparation was led by V.T., N.B., T.K., Y.N. and G.R., with contributions from all authors.

## Conflict of interest

The authors declare no conflict of interest.

1

## Supporting information

As a service to our authors and readers, this journal provides supporting information supplied by the authors. Such materials are peer reviewed and may be re‐organized for online delivery, but are not copy‐edited or typeset. Technical support issues arising from supporting information (other than missing files) should be addressed to the authors.

Supporting Information

## Data Availability

The data that support the findings of this study are available from the corresponding author upon reasonable request.
