# Supplementary material for: CeO2 Supported Gold Nanocluster Catalysts for CO Oxidation: Surface Evolution Influenced by the Ligand Shell
Source: ChemCatChem. 2022 May 18;14(14):e202200322. doi: 10.1002/cctc.202200322 (PMC9400996; doi:10.1002/cctc.202200322)
Supplement: Supplementary file 1 — Supporting Information [file CCTC-14-0-s001.pdf]

ChemCatChem

Supporting Information

CeO_2 Supported Gold Nanocluster Catalysts for CO Oxidation: Surface Evolution Influenced by the Ligand Shell

Vera Truttmann, Hedda Drexler, Michael Stöger-Pollach, Tokuhisa Kawawaki, Yuichi Negishi, Noelia Barrabés,* and Günther Rupprechter

Supporting Information

CeO_2 Supported Gold Nanocluster Catalysts for CO Oxidation: Surface Evolution Influenced by the Ligand Shell

Vera Truttmann,[1] Hedda Drexler,[1] Michael Stöger-Pollach,[2] Tokuhisa Kawawaki,[3] Yuichi Negishi,[3] Noelia Barrabés [1,]* and Günther Rupprechter [1]

[1] Institute of Materials Chemistry, TU Wien, Getreidemarkt 9/165, 1060 Vienna, Austria
[2] University Service Center for Transmission Electron Microscopy (USTEM), TU Wien, Wiedner Hauptstraße 8-10, 1040 Vienna, Austria
[3] Department of Applied Chemistry, Faculty of Science, Tokyo University of Science, Kagurazaka, Shinjuku-ku, Tokyo 162-8601, Japan

* Correspondence: noelia.rabanal@tuwien.ac.at

1. Synthesis and Characterization of Au Nanoclusters

The gold nanoclusters were synthesized according to standard procedures, as described in the following. Ultraviolet-visible spectroscopy (UV-Vis) of the nanoclusters in solution (DCM, toluene) was performed on a UV-1600PC spectrometer (VWR collection).
For electrospray ionization-time of flight mass spectrometry (ESI-TOFMS), the samples were dissolved in 100% ACN, filtered through 0.2 µm PTFE filters (Ref: 4552T, Pall Corporation) and directly injected into the HPLC system (1290 Infinity II UPLC, Agilent Technologies). 5 µL of the sample was introduced into a flow of 0.075 mL min^{-1} with the eluent mixture of 10% A (99.9% H_2O, 0.1% formic acid) and 90% B (99.9% ACN, 0.1% formic acid), passing to the ESI-source over a short flow path (PEEK-capillaries). Mass spectra were recorded on an electrospray ionization-time of flight mass spectrometer (ESI-TOFMS, 6230 B, Agilent Technologies). The TOFMS settings can be found elsewhere.[1] In brief, the dual AJS ESI-source was set to positive mode with the following settings: gas temperature 200 °C, 10 L min^{-1} gas flow, sheath gas temperature 350 °C, sheath gas flow 11 L min^{-1}, and nebulizer pressure 35 psig. The fragmentor voltage and the capillary voltages were set to 180 V and 3500 V, respectively.
The TOF instrument is specified to an accuracy of ± 1 ppm and a resolution of 10000 at m/z 322. Prior to the measurement, the TOF was calibrated within the mass range of m/z 100 and the upper calibration limit of m/z 2722, and during the entire analysis the reference masses were enabled to ensure the specified values. The measurements were performed in the 1 GHz mode (m/z range: 100–20000) with a scan rate of 2.00. Background subtraction was performed via the software Agilent MassHunter Qualitative Analysis Software (B.10.00).
Matrix-assisted laser desorption ionization (MALDI) mass spectrometric spectra were obtained with a MALDI-7090 MALDI-TOF-MS mass spectrometer (Shimadzu). *trans*-2-[3-(4-tert-Butylphenyl)-2-methyl-2-propenylidene]-malononitrile (DCTB) was used as matrix. Spectra were obtained as an average of 500 profiles of single pulses at 50 Hz and with 100 µm spot size. To avoid too much fragmentation, the laser power was reduced as much as possible.
Attenuated total reflection Fourier-transform infrared spectroscopy (ATR-FTIR) of the solid cluster samples was conducted with a Perkin Elmer Spektrum 400 instrument.

1.1. $Au_{11}(PPh_3)_7Cl_3$

$Au_{11}(PPh_3)_7Cl_3$ ("Au_{11}") was synthesized following a protocol by McKenzie *et al.*[2] with minor adaptions. To a solution of $Au(PPh_3)Cl$ (400 mg) in 20 ml tetrahydrofuran (THF), 152 mg $NaBH_4$ in 20 ml ethanol (EtOH). were added. After stirring at room temperature (2 h), the red-brown crude product was precipitated in 400 mL pentane (over 2 h). The clusters were filtered and washed four times with hexane (6 ml each) and a 50:50 mixture dichloromethane/hexane (10 ml each). Due a lot of dark brown precipitate, the crude product was subsequently rinsed with THF until the filtrate was colorless. Only red precipitate remained at that point, which was redissolved in dichloromethane (DCM) (in 5 ml portions) and the solvent removed under reduced pressure.
The purified $Au_{11}(PPh_3)_7Cl_3$ was characterized by UV-Vis spectroscopy, ESI mass spectrometry and ATR-FTIR spectroscopy (Figure S1). The ESI-TOF mass spectrum (Figure S1b) shows peaks corresponding to the two known Au_{11} isomers, $Au_{11}(PPh_3)_7Cl_3$ and $[Au_{11}(PPh_3)_8Cl_2]Cl$, as well as several products of in-source fragmentation. However, the shape and positions of the maxima of the UV-Vis spectrum in Figure S1a are in perfect agreement with those reported by McKenzie *et al* [2] for $Au_{11}(PPh_3)_7Cl_3$. Considerable percentages of $[Au_{11}(PPh_3)_8Cl_2]Cl$ in the sample should lead to shifts in the UV-Vis bands.[2] Thus, the presence of $[Au_{11}(PPh_3)_8Cl_2]^+$ in the mass spectrum is attributed to be mainly a result of gas phase chemistry during the measurement. Note that the intrinsic positive charge of $[Au_{11}(PPh_3)_8Cl_2]Cl$ compared to the neutral $Au_{11}(PPh_3)_7Cl_3$ is also expected to have an influence.

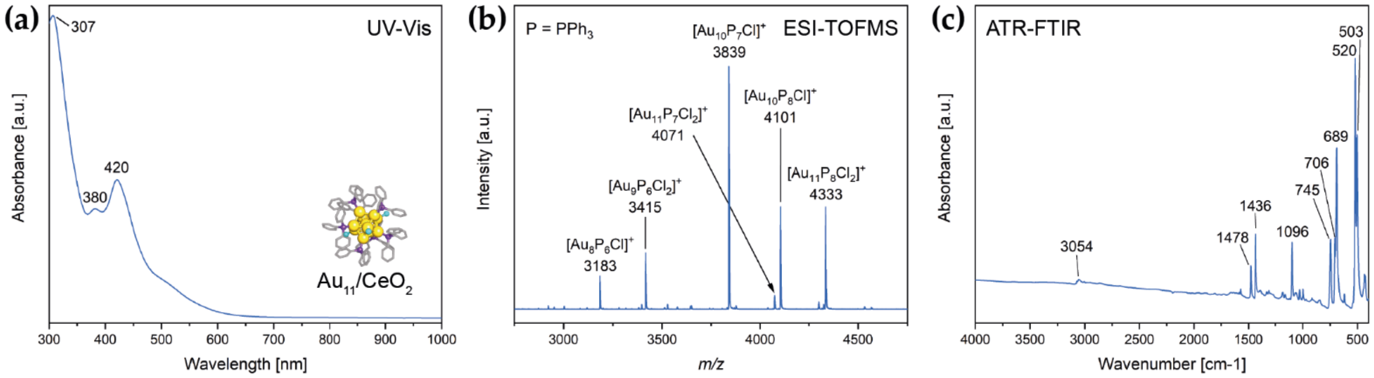

Figure S1. UV-Vis (**a**), ESI-MS (**b**) and ATR-FTIR (**c**) spectrum of $Au_{11}(PPh_3)_7Cl_3$.

1.2. $[Au_{25}(PPh_3)_{10}(SC_2H_4Ph)_5Cl_2]Cl_2$

The synthesis of $[Au_{25}(PPh_3)_{10}(SC_2H_4Ph)_5Cl_2]Cl_2$ ("Biico Au_{25}") was conducted as described previously.[3] Briefly, 7.6 mg $NaBH_4$ were added to a solution of $Au(PPh_3)Cl$ (98.5 mg) in 5.5 ml ethanol and the reaction mixture stirred at room temperature for 2 h. Afterwards, the solvent was removed under reduced pressure and the crude product washed with water, hexane and chloroform/hexane (3:2 and 1:1, 2 x 5 ml each). The precursor clusters were then obtained by extraction with chloroform. For transformation to $[Au_{25}(PPh_3)_{10}(SC_2H_4Ph)_5Cl_2]Cl_2$, 20 mg of the precursor were dissolved in 30 ml chloroform and 41 µl of 2-phenylethanethiol (2-PET) added. The mixture was stirred at room temperature for 20 h, after which the solvent was removed. The product was washed repeatedly with hexane and then extracted with EtOH, yielding $[Au_{25}(PPh_3)_{10}(SC_2H_4Ph)_5Cl_2]Cl_2$, as confirmed by UV-Vis spectroscopy, ESI mass spectrometry and ATR-FTIR spectroscopy (Figure S2).

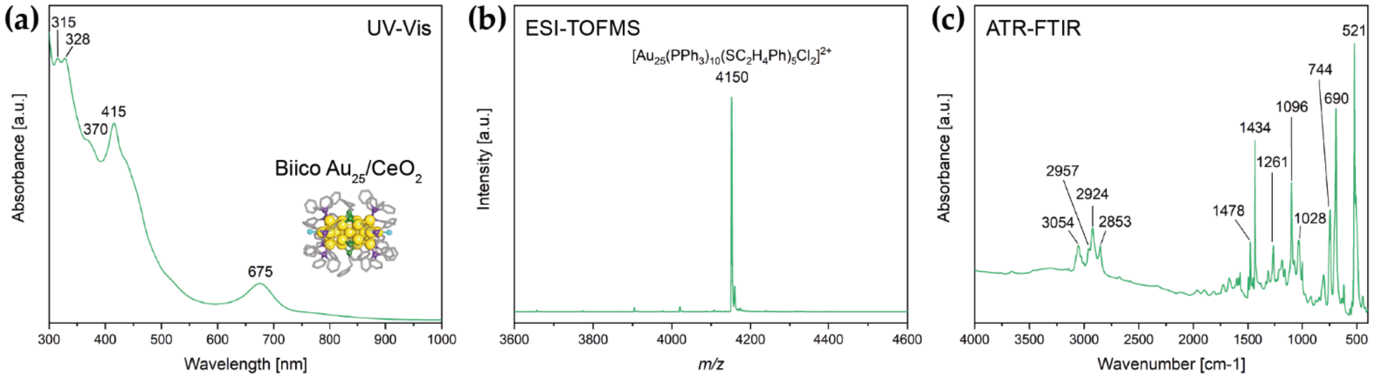

Figure S2. UV-Vis (**a**), ESI-MS (**b**) and ATR-FTIR (**c**) spectrum of $[Au_{25}(PPh_3)_{10}(SC_2H_4Ph)_5Cl_2]Cl_2$.

1.2. $[Au_{25}(SC_2H_4Ph)_{18}]^{-}TOA^{+}$

$[Au_{25}(SC_2H_4Ph)_{18}]^{-}TOA^{+}$ ("Au_{25}") was obtained according to a procedure by Shivhare *et al.*[4] 500 mg of $HAuCl_4 \cdot 3\,H_2O$ and 833 mg of tetraoctylammonium bromide (TOAB) were dissolved in 50 ml THF. 850 µl 2-PET were added, leading the color of the orange solution gradually fading out over 1 h. Subsequently, the mixture was reduced by addition of 480 mg $NaBH_4$ in 10 ml ice-cold H_2O. The reaction was continued for 4 days at room temperature. Then, the solvent was evaporated and the precipitate washed repeatedly with H_2O/methanol (1:1) and methanol (MeOH). The crude product was then extracted with acetone and purified by size exclusion chromatography (Bio-Beads S-X1 in THF). The UV-Vis, MALDI-MS and ATR-FTIR spectra of $[Au_{25}(SC_2H_4Ph)_{18}]^{-}TOA^{+}$ are shown in Figure S3. Due to the strong laser power of the mass spectrometer, the main peak in the mass spectrum in Figure S3b originates from the $[M–(Au(SC_2H_4Ph))_4]^{+}$ fragment, a typical fragment ion of Au_{25} clusters in MALDI-MS.[5]

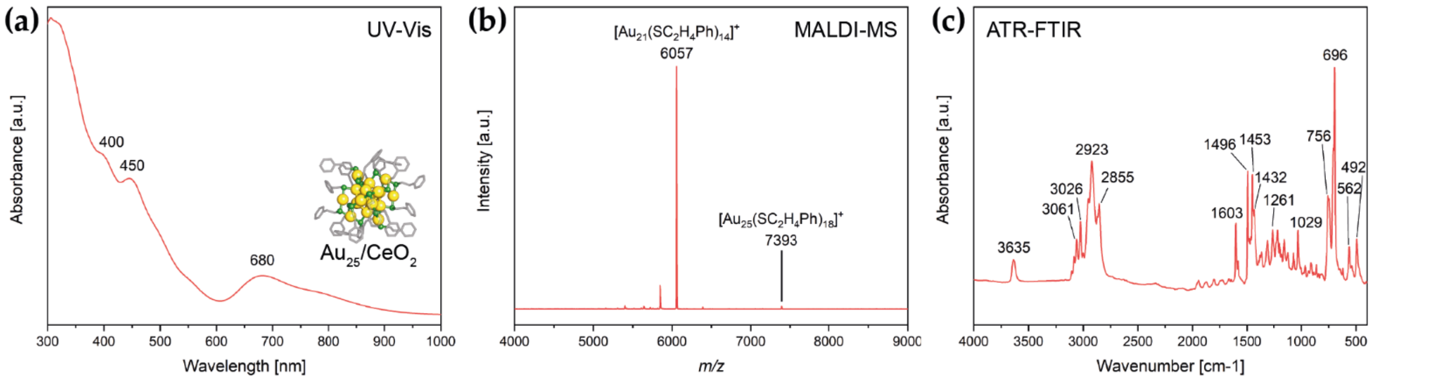

Figure S3. UV-Vis (**a**), MALDI-MS (**b**) and ATR-FTIR (**c**) spectrum of $[Au_{25}(SC_2H_4Ph)_{18}]^{-}TOA^{+}$.

2. Preparation of Ceria Supported Au Nanocluster Catalysts

The gold nanoclusters were subsequently supported on CeO_2 (Alfa Aesar, 15-30 nm particle size, 30-50 m^2/g surface area). The clusters were dissolved in toluene (Au_{25}) or MeOH (Au_{11}, Biico Au_{25}) and added dropwise to a stirred suspension of ceria in the respective solvent. After complete addition, stirring at room temperature was continued for 6 h. The supported clusters were then separated from the solution by filtration, the powder dried under reduced pressure and then grinded.

The exact metal loading (wt%) of the gold nanocluster catalysts was determined by total X-ray fluorescence spectroscopy (TXRF), using an ATOMIKA 8030C X-ray fluorescence analyzer. Samples were attached to total reflecting quartz reflectors using 1 mg of sample mixed with 5 µl of 1% poly-vinyl alcohol solution (for fixation). Blank measurements of the unloaded reflectors were performed prior to each specimen measurement to avoid cross contamination. Detection limits of the quantified elements (Au and Ce) are in the range of 10-100 µg/g.

3. Thermogravimetric Analysis/Differential Scanning Calorimetry

Simultaneous thermogravimetric analysis (TGA)/differential scanning calorimetry (DSC) was carried out using a Netzsch STA 409 PC/PG Luxx thermal analyzer. Approximately 10 mg of unsupported clusters were transferred to an Al_2O_3 crucible as a concentrated suspension in dichloromethane and dried at room temperature for 24 h to ensure complete evaporation of the solvent. The sample was subsequently transferred to the thermal analyzer and heated to 100 °C with a 5 °C/min ramp. It was kept at this temperature for 30 min before being heated to 400 °C (also 5 °C/min and 30 min holding period). The entire heating process was performed with a total gas flow of 50 ml/min of 5% O_2 in He (i.e., the pretreatment atmosphere).

It should be noted that quantitative errors of up to 10% apply to the mass loss of all samples and thus, both the TGA and the DSC signals are only interpreted qualitatively. The error is due to the small amounts of sample used (10 mg) or to traces of gold removed from the crucible during the oxidation of the ligand sphere. Moreover, in lieu of the dynamic nature of oxide supported Au nanoclusters, considering for example potential ligand migration to the support or Au sintering, it should be noted beforehand that TGA and DSC results of unsupported clusters are probably not directly transferable to their immobilized counterparts.

The main mass loss of unsupported $Au_{11}(PPh_3)_7Cl_3$ (Figure S4) occurs between 200 °C and 300 °C, with a gradual on-set starting at approximately 150 °C. In addition, the DSC curve shows a sharp negative peak at 157 °C, which could indicate a structural rearrangement process. Further small peaks in the DSC curve are observed at 189 °C and 296 °C, which are approximately at the beginning and end of the period of main mass loss. Overall, the observed TGA curve agrees with the temperature programmed oxidation measurements of the CeO_2 supported clusters catalysts, which showed CO_2 formation above 200 °C (see Figure 3 and Figure S10). It also indicates that under the pretreatment conditions applied herein, a temperature of 300 °C is necessary to remove all ligands and/or their residues from the unsupported clusters, which correlates with the pronounced increase in reactivity for the 300 °C pretreated catalyst (Figure 2a). Compared to TGA of $[Au_{11}(PPh_3)_8Cl_2]Cl$ reported by Leong and coworkers,[6] the mass loss proceeds and extends to higher temperatures, which may be related to differences in experimental conditions.

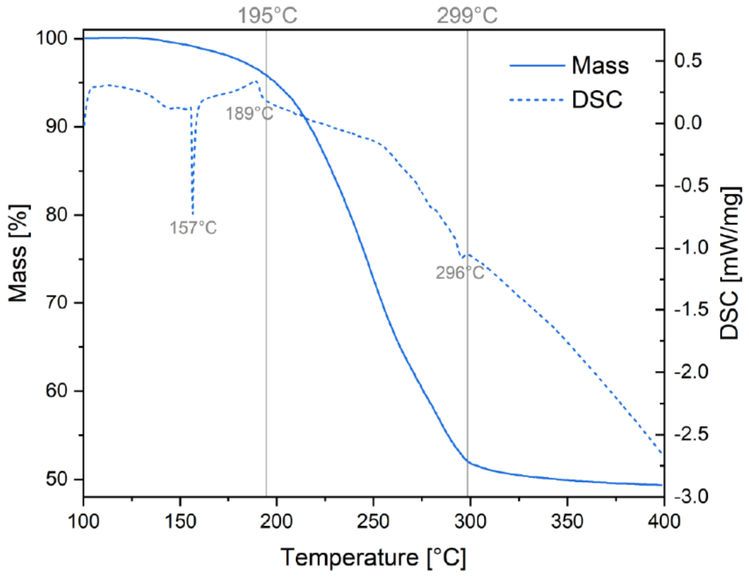

Figure S4. Mass loss (solid line) and differential scanning calorimetric signal (dashed line) of $Au_{11}(PPh_3)_7Cl_3$ at pretreatment conditions.

For $[Au_{25}(PPh_3)_{10}(SC_2H_4Ph)_5Cl_2]Cl_2$, (Figure S5), mass loss starts at around 165 °C and continues up to 370 °C, though the loss is marginal above 263 °C. It is accompanied by several negative peaks and small dips in the DSC curve at 165 °C, 192 °C, 244 °C, ≈270 °C and ≈350 °C. This could indicate several changes to the initial cluster structure during the ligand detachment process. Notably, the major ligand removal process is still ongoing at 250 °C and also structural changes seem to occur even above 250 °C, which may contribute to the sudden jump in activity after pretreatment at 300 °C (cf. Figure 2b). The mass loss curve of the unsupported clusters also compares well to the CO_2 formation and O_2 consumption peaks observed in the temperature programmed oxidation experiments with CeO_2 supported catalyst (see Figure 3 and Figure S10), which showed an on-set at 165-175 °C and a maximum at 267 °C. Zhu *et al.*[7] also performed TGA of $[Au_{25}(PPh_3)_{10}(SC_2H_4Ph)_5Cl_2]Cl_2$ nanoclusters and observed beginning mass loss at 150 °C, which continued up to ~300 °C. They also reported that the thiolate ligands were removed first, followed by the phosphines and chlorine ligands.

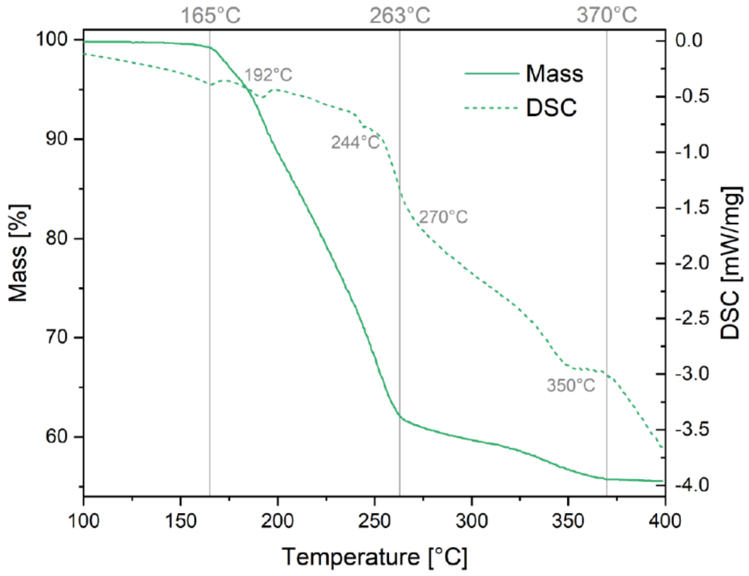

Figure S5. Mass loss (solid line) and differential scanning calorimetric signal (dashed line) of $[Au_{25}(PPh_3)_{10}(SC_2H_4Ph)_5Cl_2]Cl_2$ at pretreatment conditions.

Ligand desorption of $[Au_{25}(SC_2H_4Ph)_{18}]^-TOA^+$ started at ≈174 °C, accompanied by a negative DSC peak at 176 °C. The mass loss then occurs up to 298 °C, though most part of the ligand sphere seems already removed when reaching 240 °C. In addition, also DSC only shows significant features below 240 °C. This indicates that most structural changes occur below 250 °C, which may contribute to the significantly increased activity of catalysts pretreated at that

temperature (Figure 2c). For the CeO_2 supported clusters, similar temperatures were observed by temperature programmed oxidation (Figure 3 and Figure S10): the CO_2 formation starts between 150-165 °C (slightly lower than in TGA) and peaks at 235-245 °C. Complete removal of the protecting ligands/counterions at 250-300 °C in TGA was reported by Nie *et al.*[8] and at 250 °C by Zhu *et al.*[7] However, both reported a slightly higher on-set temperature of ~190–200 °C.[7-8]

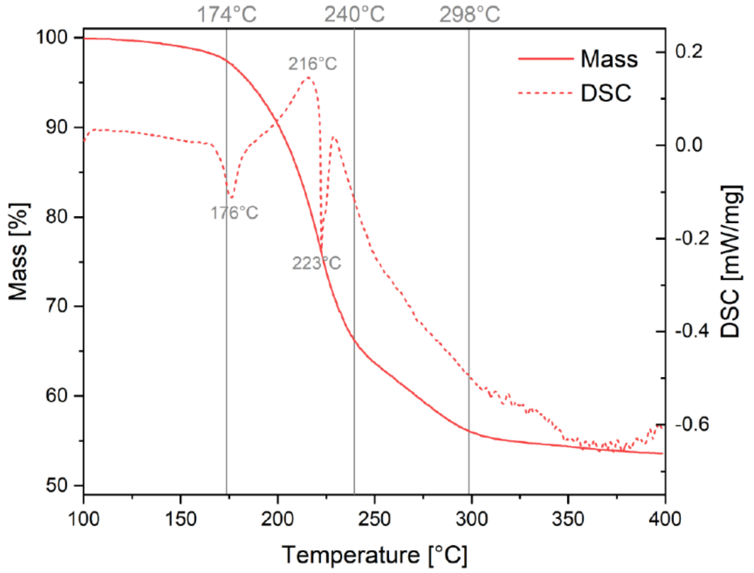

Figure S6. Mass loss (solid line) and differential scanning calorimetric signal (dashed line) of $[Au_{25}(SC_2H_4Ph)_{18}]TOA$ at pretreatment conditions.

4. Additional Kinetic Measurements

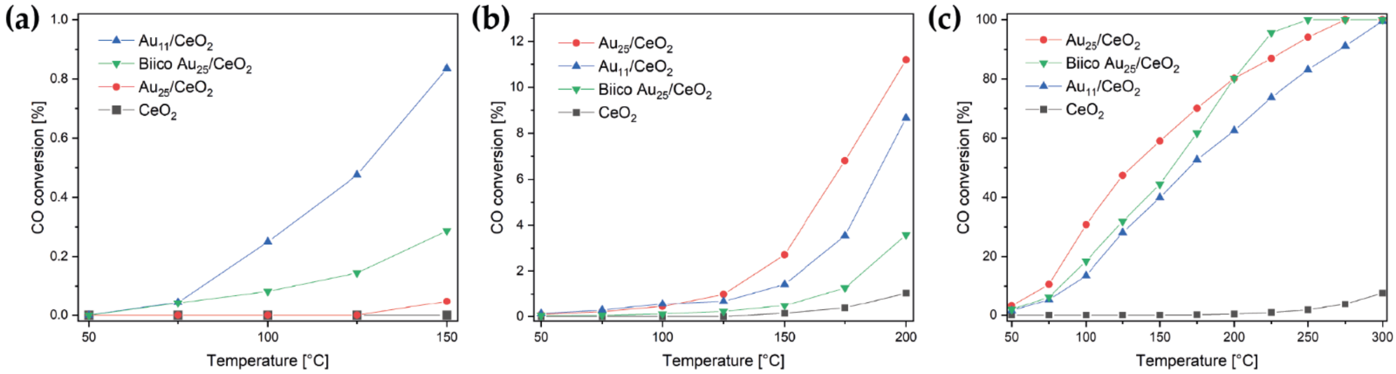

Figure S7. Comparison of the catalytic activity of nanocluster catalysts (0.3 wt% Au loading, 15 mg catalyst) pretreated at (**a**) 150 °C, (**b**) 200 °C and (**c**) 300 °C. Note that the conversion scales differ by two orders of magnitude.

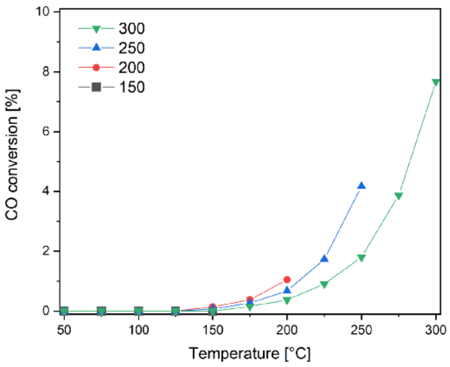

Figure S8. CO conversion of the pure CeO_2 (15 mg) pretreated at different temperatures.

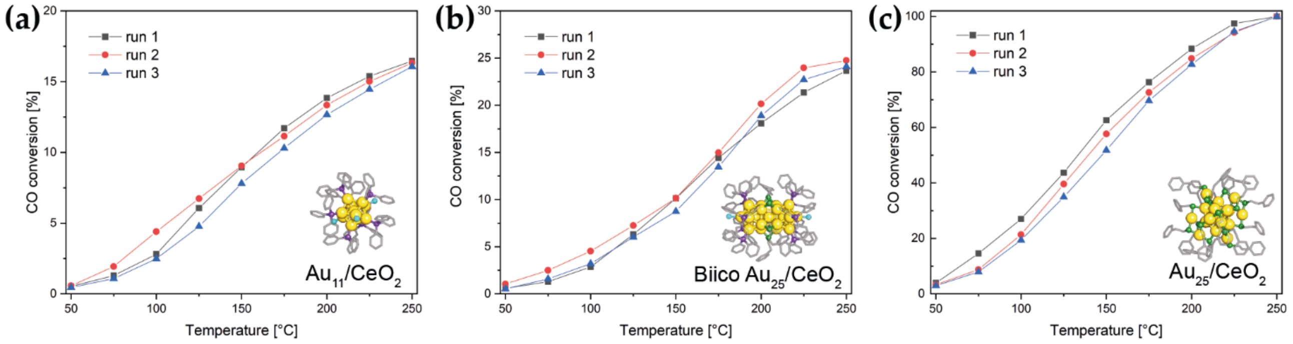

Figure S9. CO conversion of the same catalyst sample (0.3 wt% Au loading, 15 mg catalyst) in 3 consecutive CO oxidation runs after pretreatment at 250 °C: (**a**) Au_{11}/CeO_2, (**b**) Biico Au_{25}/CeO_2 and (**c**) Au_{25}/CeO_2.

5. Additional Temperature Programmed Oxidation Spectra

In addition to the CO_2 generation and O_2 consumption spectra obtained for a 250 °C pretreatment during the *in situ* transmission infrared studies, a separate temperature programmed oxidation (TPO) experiment until 300 °C was performed. A reactor set-up comparable to the one used for the catalytic studies (see experimental section in the main manuscript) was employed. ~23 mg of sample was placed in a quartz tube between glass wool plugs and heated to 300 °C (10 °C/min) in an atmosphere of 5% O_2 in Ar (50 ml/min total gas flow). The sample was kept at 300 °C for 30 min. The exhaust gas composition was analyzed by mass spectrometry (Pfeiffer Vacuum QME 200 with SEM detector).

Figure S10 shows the CO_2 evolution (a) and O_2 consumption (b) spectra of the different catalysts during pretreatment till 300 °C. As seen in Figure S10a, CO_2 desorption from CeO_2 was observed from about 70-130 °C for all samples.[8] For Au_{25}/CeO_2, CO_2 formation started at approximately 165 °C, with the peak centered at 245 °C. A very broad peak is observed for its O_2 consumption, with an approximate on-set at 140 °C. For Biico Au_{25}/CeO_2 and Au_{11}/CeO_2, CO_2 formation set in at ~175 °C (center 267 °C) and ~200 °C (center 275 °C), respectively. Their O_2 consumption peaks were centered at 270 °C (Biico Au_{25}/CeO_2) and 275 °C (Au_{11}/CeO_2). Pure CeO_2 also showed CO_2 evolution and very minor O_2 consumption above 200 °C.

It should be noted that the disturbance in the low-temperature region of the O_2 consumption spectra (Figure S10b) of Au_{11}/CeO_2 and Au_{25}/CeO_2 are mainly due to a negative spike in the argon carrier gas pressure, which can also be seen in the CO_2 generation spectra (Figure S10a). The distortions of the Biico Au_{25}/CeO_2 spectrum originate from the O_2 mass flow controller.

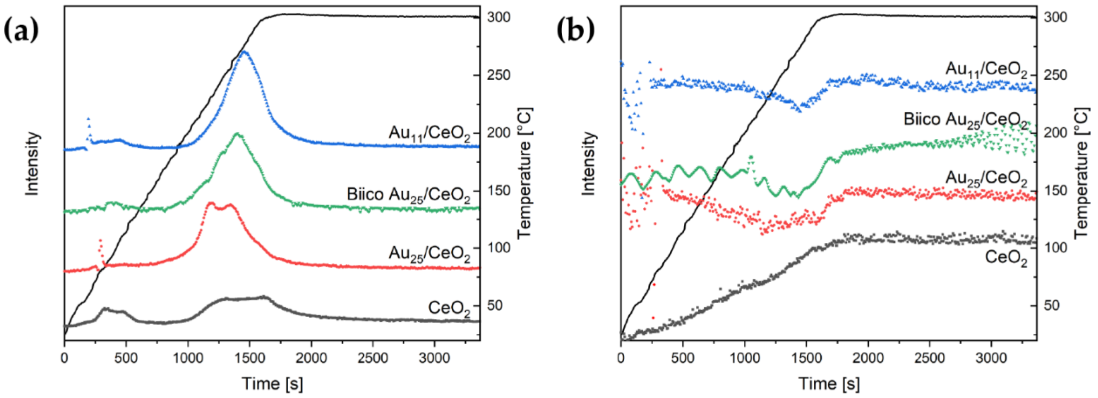

Figure S10. CO_2 generation (**a**) and O_2 consumption spectra (**b**) of the different catalysts during oxidative pretreatment up to 300 °C. Au content in catalyst: 1.2 wt% for all cluster catalysts. Spectra were normalized by the carrier gas signal to compensate for changes in pressure. Spectra are offset for better visibility.

6. Additional Spectra of *In Situ* Transmission Infrared Measurements

6.1 Catalytic CO Conversion

Figure S11 shows the CO conversion (after 250 °C pretreatment) during the *operando* IR measurements determined by gas chromatography. To facilitate a more meaningful comparison of the IR spectra, similar conversion levels (activity) of the clusters catalysts was aimed for. Therefore, in case of Au_{11}/CeO_2 and Biico Au_{25}/CeO_2 catalysts, samples with 1.2 wt% Au loading instead of 0.3 wt% were employed (Au_{25}/CeO_2 still had 0.3 wt%). For all catalysts, a thin wafer with ~10 mg mass was produced. To show that the activity is similar to that observed in the kinetic experiments with the powdered catalyst in a quartz tube, the CO conversion was normalized to 0.3 wt% and 15 mg catalyst, (Figure S11b). Small deviations can be attributed to the different experimental setup (pellet *vs.* powder).

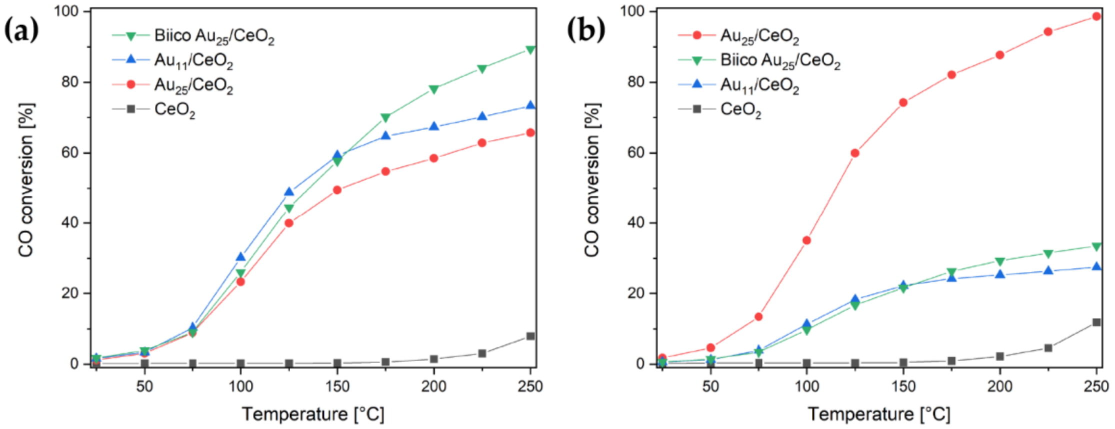

Figure S11. CO conversion during *operando* transmission IR studies: (**a**) per 10 mg catalyst pellet (Au content in catalyst: Au_{11}/CeO_2 and BiicoAu_{25}/CeO_2: 1.2wt%, Au_{25}/CeO_2: 0.3 wt%) and (**b**) normalized to 0.3 wt% Au and 15 mg catalyst (*cf.* kinetic studies). Pretreatment at 250 °C at 5% O_2 in He.

6.2 Additional Infrared Spectra of the Oxidative Pretreatment

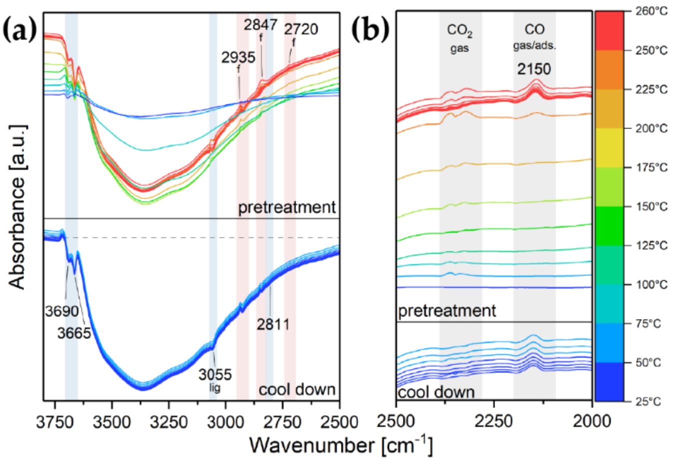

Figure S12. Difference spectra of Au_{11}/CeO_2 during oxidative pretreatment: (**a**) 3800–2500 cm^{-1} and (**b**) 2500–2000 cm^{-1}. Bands decreasing during the pretreatment are indicated by a light blue background color and marked at the bottom, increasing ones by a light red one and marked on top. Assigned species are indicated by abbreviations: f = formates, lig = ligands. The spectrum of the as-prepared catalyst in He at RT was used as background. Au content in catalyst: 1.2 wt%.

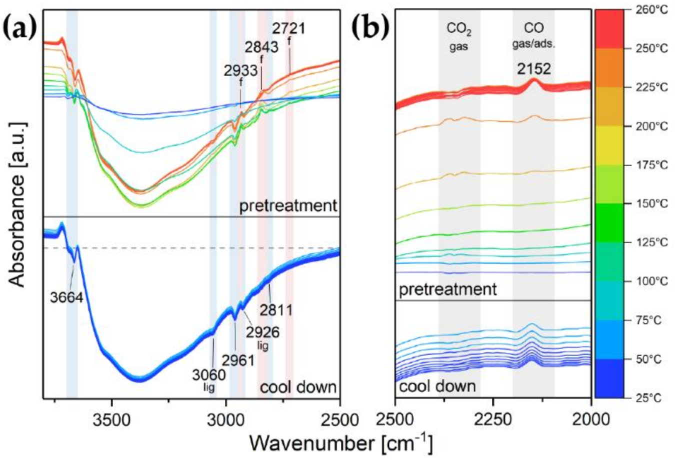

Figure S13. Difference spectra of Biico Au_{25}/CeO_2 during oxidative pretreatment: (**a**) 3800–2500 cm^{-1} and (**b**) 2500–2000 cm^{-1}. Bands decreasing during the pretreatment are indicated by a light blue background color and marked at the bottom, increasing ones by a light red one and marked on top. Assigned species are indicated by abbreviations: f = formates, lig = ligands. The spectrum of the as-prepared catalyst in He at RT was used as background. Au content in catalyst: 1.2 wt%.

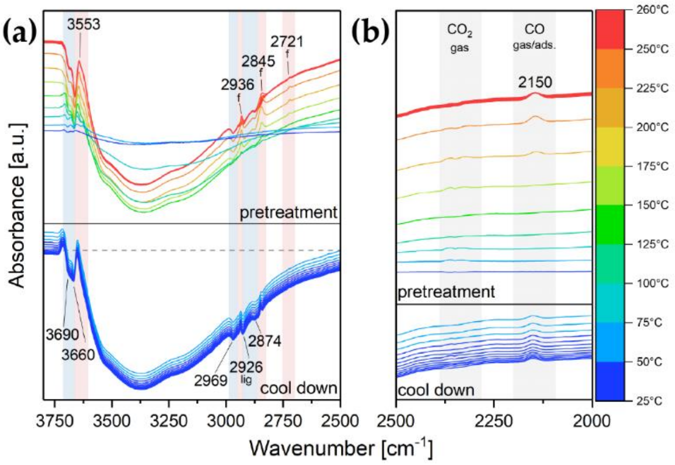

Figure S14. Difference spectra of Au_{25}/CeO_2 during oxidative pretreatment: (**a**) 3800–2500 cm^{-1} and (**b**) 2500–2000 cm^{-1}. Bands decreasing during the pretreatment are indicated by a light blue background color and marked at the bottom, increasing ones by a light red one and marked on top. Assigned species are indicated by abbreviations: f = formates, lig = ligands. The spectrum of the as-prepared catalyst in He at RT was used as background. Au content in catalyst: 0.3 wt%.

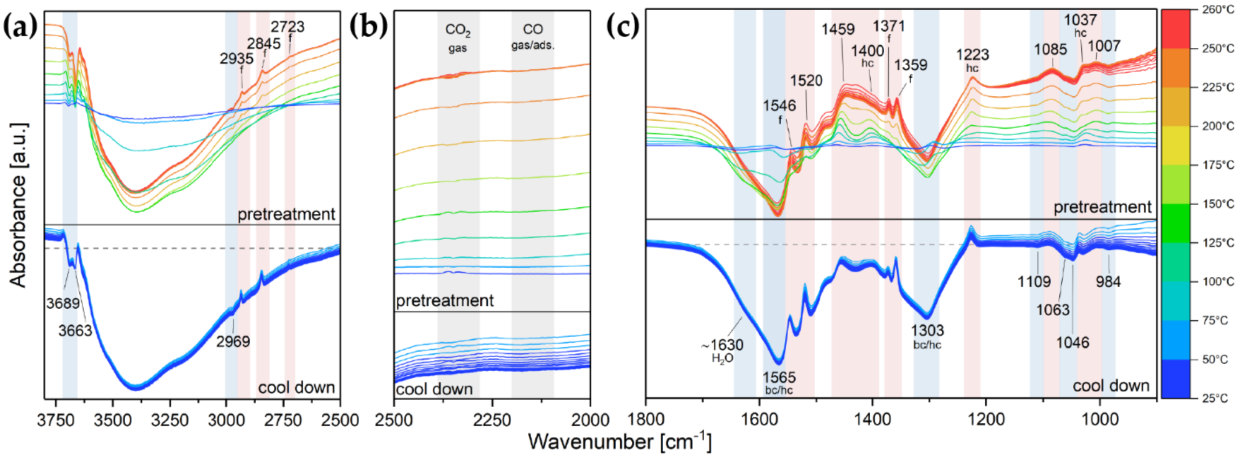

Figure S15. Difference spectra of the CeO_2 support during oxidative pretreatment: (**a**) 3800–2500 cm^{-1}, (**b**) 2500–2000 cm^{-1} and (**c**) 1800–900 cm^{-1}. Bands decreasing during the pretreatment are indicated by a light blue background color and marked at the bottom, increasing ones by a light red one and marked on top. Assigned species are indicated by abbreviations: f = formates, hc = hydrogen carbonates, bc/tc = bidentate/tridentate carbonates. The spectrum of CeO_2 in He at RT was used as background.

6.3 Additional Spectra of the CO Oxidation

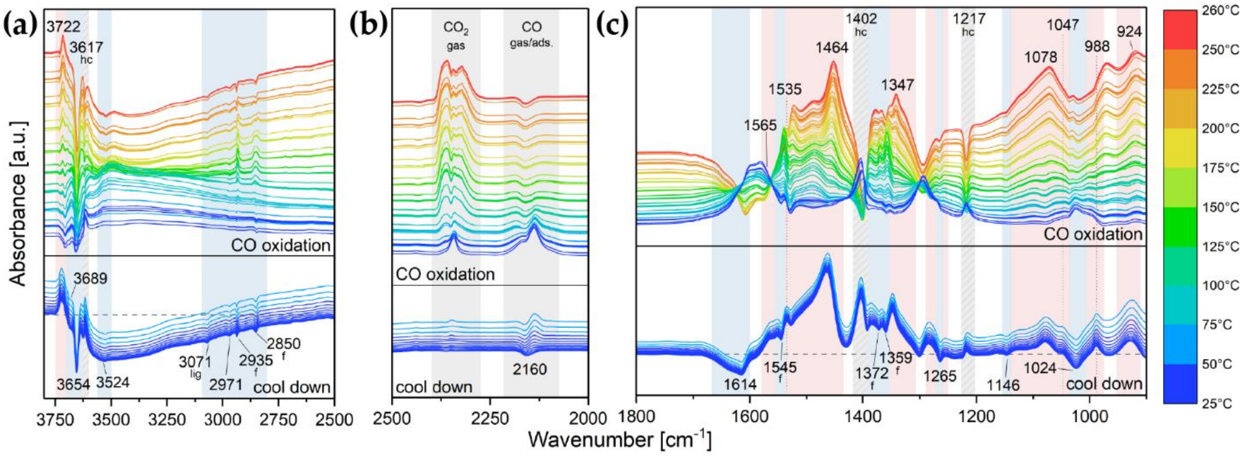

Figure S16. Difference spectra of Biico Au_{25}/CeO_2 during CO oxidation: (**a**) 3800–2500 cm^{-1}, (**b**) 2500–2000 cm^{-1} and (**c**) 1800–900 cm^{-1}. Bands decreasing during the pretreatment are indicated by a light blue background color and marked at the bottom, increasing ones by a light red one and marked on top. Bands decreasing during reaction but restored upon cool down are indicated by a grey shaded background. Assigned species are indicated by abbreviations: f = formates, hc = hydrogen carbonates, lig = ligands. The spectrum of the pretreated catalyst after the CO adsorption experiment in He at RT was used as background. Au content in catalyst: 1.2 wt%.

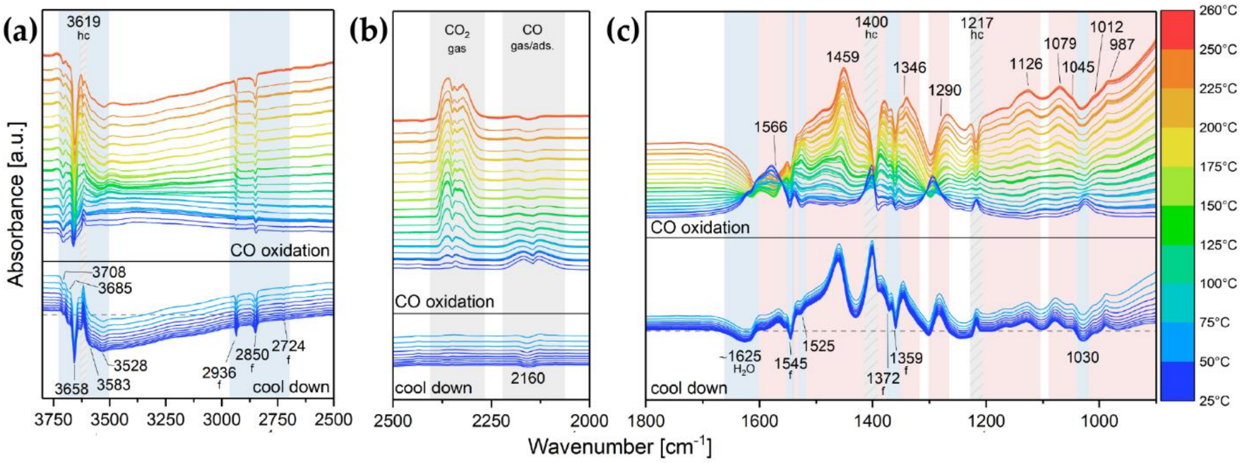

Figure S17. Difference spectra of Au_{25}/CeO_2 during CO oxidation: (**a**) 3800–2500 cm^{-1}, (**b**) 2500–2000 cm^{-1} and (**c**) 1800–900 cm^{-1}. Bands decreasing during the pretreatment are indicated by a light blue background color and marked at the bottom, increasing ones by a light red one and marked on top. Bands decreasing during reaction but restored upon cool down are indicated by a grey shaded background. Assigned species are indicated by abbreviations: f = formates, hc = hydrogen carbonates. The spectrum of the pretreated catalyst after the CO adsorption experiment in He at RT was used as background. Au content in catalyst: 0.3 wt%.

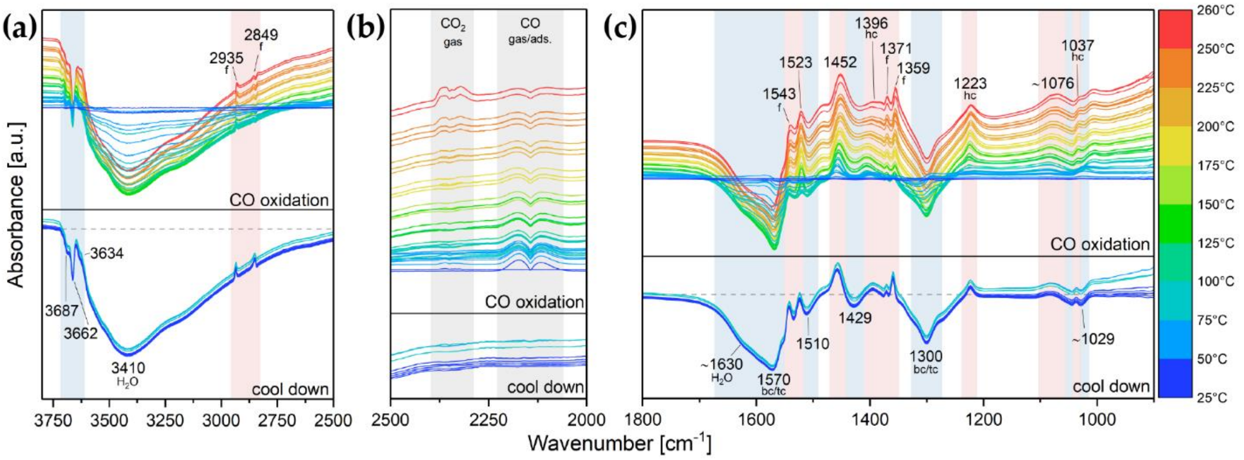

Figure S18. Difference spectra of the CeO_2 support during CO oxidation: (**a**) 3800–2500 cm^{-1}, (**b**) 2500–2000 cm^{-1} and (**c**) 1800–900 cm^{-1}. Bands decreasing during the pretreatment are indicated by a light blue background color and marked at the bottom, increasing ones by a light red one and marked on top. Assigned species are indicated by abbreviations: f = formates, hc = hydrogen carbonates, bc/tc = bidentate/tridentate carbonates. The spectrum of CeO_2 after the CO adsorption experiment in He at RT was used as background.

6.4 Infrared Spectra of Post-Reaction CO Adsorption

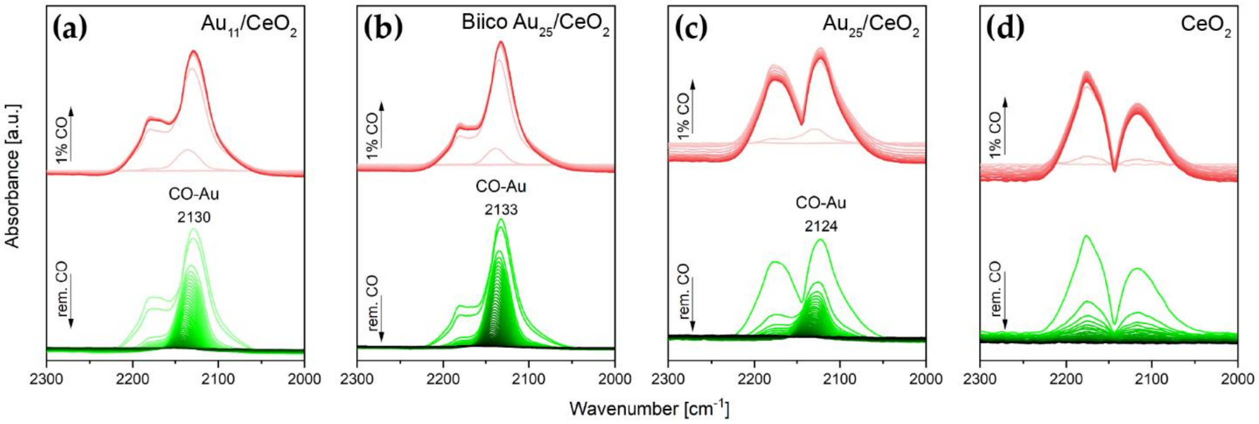

Figure S19. Transmission infrared spectra of room temperature CO adsorption on the used catalysts (250 °C pretreatment and reaction): (**a**) Au_{11}/CeO_2 (**b**) Biico Au_{25}/CeO_2, (**c**) Au_{25}/CeO_2 and (**d**) CeO_2. The red spectra were obtained during exposure of the sample to an atmosphere of 1% CO in He (50 ml/min total gas flow), the green spectra upon removal of gas phase CO by flowing 50 ml/min He. Au content in catalyst: Au_{11}/CeO_2 and Biico Au_{25}/CeO_2: 1.2 wt%, Au_{25}/CeO_2: 0.3 wt%.

6.5 Additional Infrared Spectra at Different Steps of the Reaction

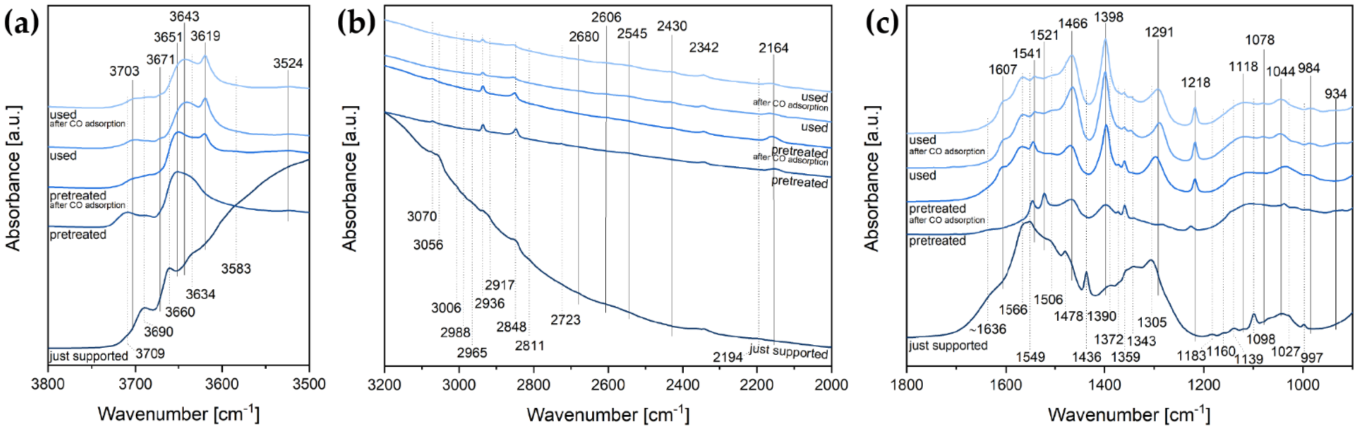

Figure S20. Comparison of infrared absorbance spectra of Au_{11}/CeO_2 in He at room temperature at different steps of the catalytic process: (**a**) 3800–3500 cm^{-1}, (**b**) 3200–2000 cm^{-1} and (**c**) 1800–900 cm^{-1}. Decreasing bands are indicated by dotted lines and marked on the bottom, increasing ones by solid lines and marked on top. Au content in catalyst: 1.2 wt%.

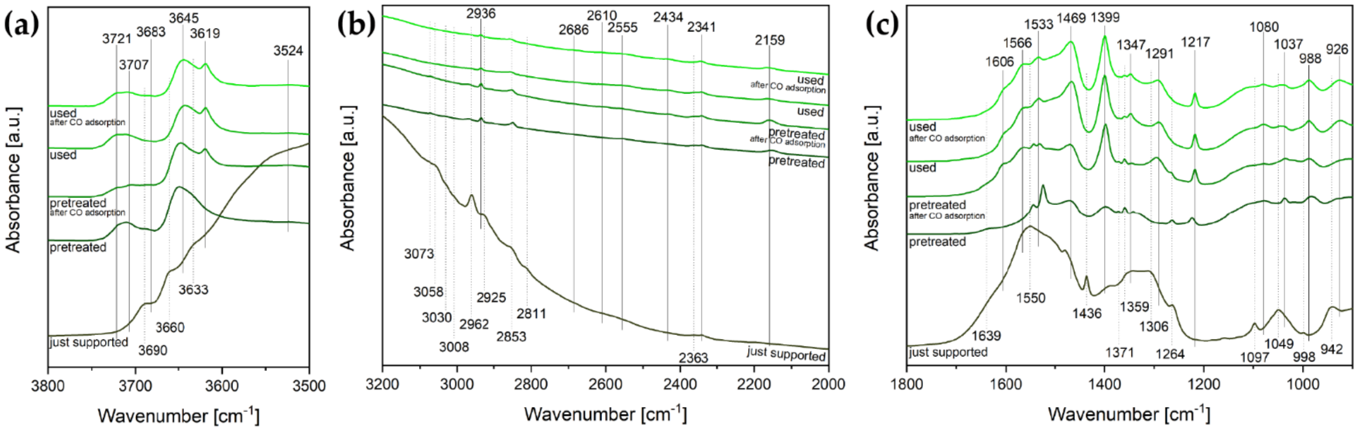

Figure S21. Comparison of infrared absorbance spectra of Biico Au_{25}/CeO_2 in He at room temperature at different steps of the catalytic process: (**a**) 3800–3500 cm^{-1}, (**b**) 3200–2000 cm^{-1} and (**c**) 1800–900 cm^{-1}. Decreasing bands are indicated by dotted lines and marked on the bottom, increasing ones by solid lines and marked on top. Au content in catalyst: 1.2 wt%.

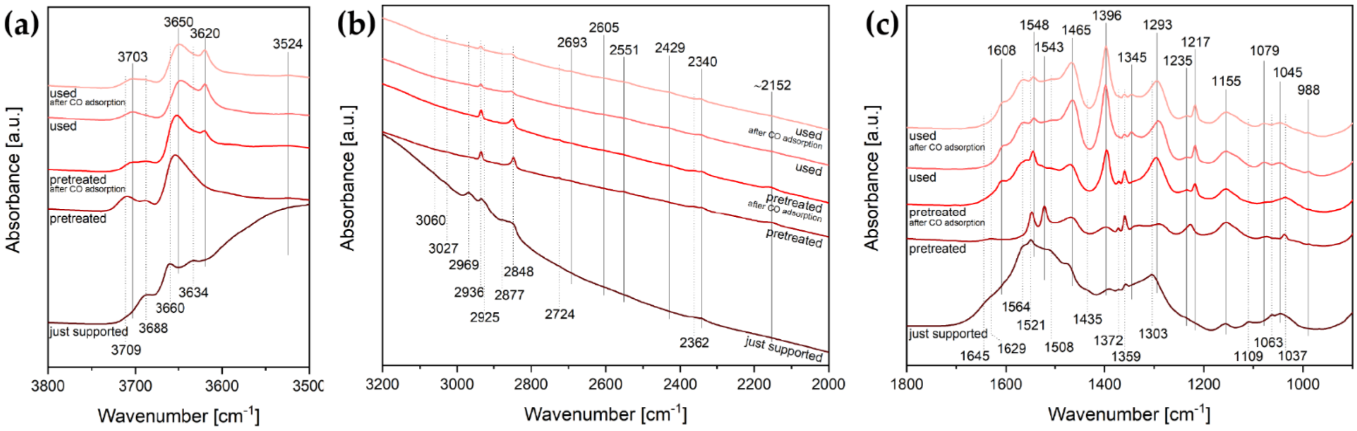

Figure S22. Comparison of infrared absorbance spectra of Au_{25}/CeO_2 in He at room temperature at different steps of the catalytic process: (**a**) 3800–3500 cm^{-1}, (**b**) 3200–2000 cm^{-1} and (**c**) 1800–900 cm^{-1}. Decreasing bands are indicated by dotted lines and marked on the bottom, increasing ones by solid lines and marked on top. Au content in catalyst: 0.3 wt%.

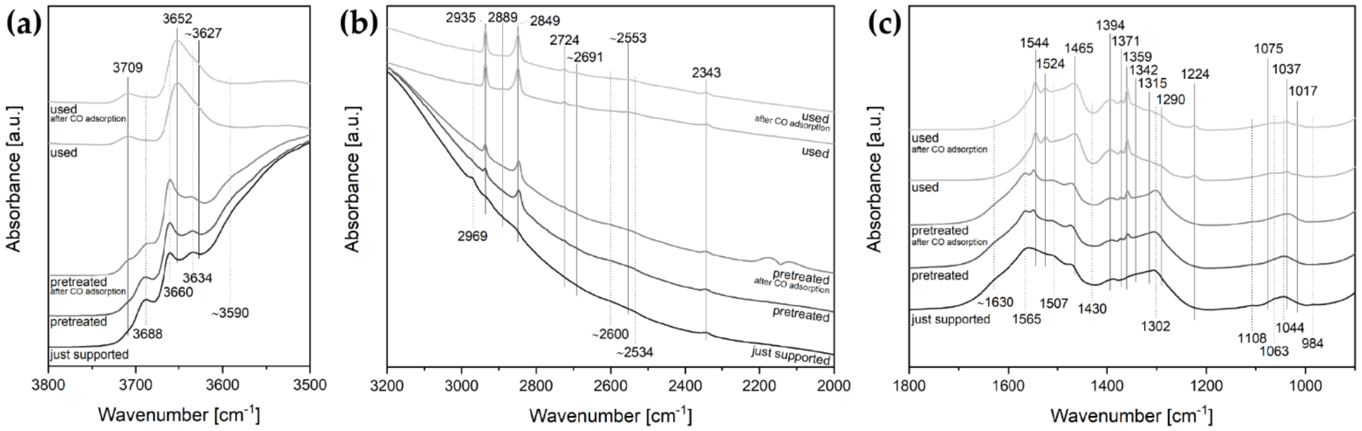

Figure S23. Comparison of infrared absorbance spectra of the CeO_2 support in He at room temperature at different steps of the catalytic process: (**a**) 3800–3500 cm^{-1}, (**b**) 3200–2000 cm^{-1} and (**c**) 1800–900 cm^{-1}. Decreasing bands are indicated by solid lines and marked on the bottom, increasing ones by solid lines and marked on top.

6.6 Bands of Adsorbate Species on CeO_2 as Stated in the Literature

Table S1. Bands (in cm^{-1}) of typical adsorbate species on CeO_2 (wavenumbers from respective literature references)

Species	Band 1	Band 2	Band 3	Band 4	Ref.
formates	ν(CH) 2850	ν(CO) 1547, 1360	δ(OCH) 1372	comb 2935, 2725	[9]
	ν(CH) 2845	ν(CO) 1599, 1553, 1542, 1362, 1248	δ(OCH) 1371	comb 2933, 2723	[10]
	ν(CH) 2853	ν(CO) 1590–1510, 1390–1350	δ(OCH) 1390–1350	comb 2950	[11]
hydrogen carbonates	ν(OH) 3616	ν(CO) 1602, 1397, 1030	δ(COH) 1218		[9]
	ν(OH) 3617	ν(CO) 1613–1599, 1413–1391, 1045–1025	δ(COH) 1218		[10]
		ν(CO) 1404	δ(COH) 1217		[12]
monodentate carbonates	ν(CO) 1504, 1351				[10]
	ν(CO) 1507				[12]
mono- or polydentate carbonates	ν(CO) 1473, 1361				[11]
	ν(CO) 1471, 1396				[13]
bidentate carbonates	ν(CO) 1732–1722, 1147–1136				[9]
	ν(CO) 1567, 1289, 1014				[10]
	ν(CO) 1570, 1287, 1011				[12]
bi- or tridentate carbonates	ν(CO) 1570, 1296				[11]
	ν(CO) 1600, 1293				[13]
bridged carbonates	ν(CO) 1736, 1135				[10]
tridentate carbonates	ν(CO) 1618–1451, 1377–1281, 1065–990	comb 2876–2850			[9]

polydentate carbonates	ν(CO) 1462, 1353, 1066		[10]
	ν(CO) 1476, 1367		[12]
hydroxyl species	ν(OH) 3710–3600, 3510		[10]
	ν(OH) 3712, 3658, 3492		[9]
	ν(OH) 3729, 3700, 3686, 3635		[13]
sulfates	ν(SO) 1400–1340 (surface)	ν(SO) ≈1200 (broad; bulklike)	[14]
phosphates	ν(PO) 1158, 1000, 950	probably ν(POH) 1625	[15]

7. Additional High-angle Annular Dark-field Scanning Transmission Electron Microscopy

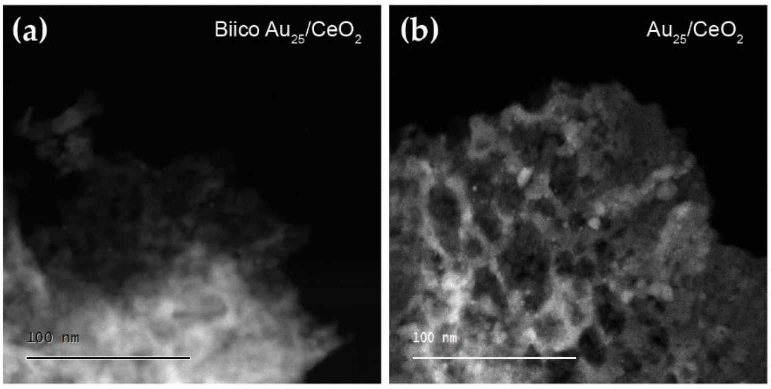

Figure S24. Overview HAADF-STEM images of Biico Au_{25}/CeO_2 (**a**) and Au_{25}/CeO_2 (**b**) after pretreatment and reaction at 250 °C.

References

[1] V. Truttmann, S. Pollitt, H. Drexler, S. P. Nandan, D. Eder, N. Barrabés and G. Rupprechter, *J. Chem. Phys.* **2021**, *155*, 161102.

[2] L. C. McKenzie, T. O. Zaikova and J. E. Hutchison, *J. Am. Chem. Soc.* **2014**, *136*, 13426-13435.

[3] a) L. V. Nair, S. Hossain, S. Takagi, Y. Imai, G. Hu, S. Wakayama, B. Kumar, W. Kurashige, D.-e. Jiang and Y. Negishi, *Nanoscale* **2018**, *10*, 18969-18979; b) Y. Shichibu, Y. Negishi, T. Watanabe, N. K. Chaki, H. Kawaguchi and T. Tsukuda, *J. Phys. Chem. C* **2007**, *111*, 7845-7847; c) G. H. Woehrle, M. G. Warner and J. E. Hutchison, *J. Phys. Chem. B* **2002**, *106*, 9979-9981.

[4] A. Shivhare, S. J. Ambrose, H. Zhang, R. W. Purves and R. W. J. Scott, *Chem. Commun.* **2013**, *49*, 276-278.

[5] A. Dass, A. Stevenson, G. R. Dubay, J. B. Tracy and R. W. Murray, *J. Am. Chem. Soc.* **2008**, *130*, 5940-5946.

[6] Y.-Z. Li and W. K. Leong, *RSC Adv.* **2019**, *9*, 5475-5479.

[7] Y. Zhu, H. Qian, A. Das and R. Jin, *Chin. J. Catal.* **2011**, *32*, 1149-1155.

[8] X. Nie, H. Qian, Q. Ge, H. Xu and R. Jin, *ACS Nano* **2012**, *6*, 6014-6022.

[9] G. N. Vayssilov, M. Mihaylov, P. S. Petkov, K. I. Hadjiivanov and K. M. Neyman, *J. Phys. Chem. C* **2011**, *115*, 23435-23454.

[10] C. Binet, M. Daturi and J.-C. Lavalley, *Catal. Today* **1999**, *50*, 207-225.

[11] A. L. Cámara, S. Chansai, C. Hardacre and A. Martínez-Arias, *Int. J. Hydrogen Energy* **2014**, *39*, 4095-4101.

[12] K. Yoshikawa, H. Sato, M. Kaneeda and J. N. Kondo, *J. CO2 Util.* **2014**, *8*, 34-38.

[13] Z. Ren, F. Peng, J. Li, X. Liang and B. Chen, *Catalysts* **2017**, *7*, 48.

[14] M. Waqif, P. Bazin, O. Saur, J. C. Lavalley, G. Blanchard and O. Touret, *Appl. Catal., B* **1997**, *11*, 193-205.

[15] Q. Dai, Z. Zhang, J. Yan, J. Wu, G. Johnson, W. Sun, X. Wang, S. Zhang and W. Zhan, *Environ. Sci. Technol.* **2018**, *52*, 13430-13437.
